# Dual targeting of the DNA damage response pathway and BCL-2 in diffuse large B-cell lymphoma

**DOI:** 10.1038/s41375-021-01347-6

**Published:** 2021-07-24

**Authors:** Alessandra Rossi, Stefania Orecchioni, Paolo Falvo, Valentina Tabanelli, Elena Baiardi, Claudio Agostinelli, Federica Melle, Giovanna Motta, Angelica Calleri, Stefano Fiori, Chiara Corsini, Beatrice Casadei, Saveria Mazzara, Umberto Vitolo, Francesco Bertolini, Pier Luigi Zinzani, Myriam Alcalay, Pier Giuseppe Pelicci, Stefano Pileri, Corrado Tarella, Enrico Derenzini

**Affiliations:** 1 Onco-Hematology Division, IEO European Institute of Oncology IRCCS, Milan, Italy; 2 Laboratory of Hematology-Oncology, IEO European Institute of Oncology IRCCS, Milan, Italy; 3 Division of diagnostic Haematopathology, IEO European Institute of Oncology IRCCS, Milan, Italy; 4 Hematopathology Unit, Department of Experimental, Diagnostic, and Specialty Medicine (DIMES), Bologna University School of Medicine, Bologna, Italy; 5 Haematopathology Unit, IRCCS Azienda Ospedaliero-Universitaria di Bologna, Bologna, Italy; 6 IRCCS Azienda Ospedaliero-Universitaria di Bologna, Institute of Hematology and Medical Oncology “L. e A. Seragnoli”, Department of Experimental, Diagnostic, and Specialty Medicine (DIMES), University of Bologna, Bologna, Italy; 7 Multidisciplinary Oncology Outpatient Clinic, Candiolo Cancer Institute, FPO-IRCCS, Candiolo, Italy; 8 Department of Experimental Oncology, IEO European Institute of Oncology IRCCS, Milan, Italy; 9 Department of Oncology and Hemato-Oncology, University of Milan, Milan, Italy; 10 Department of Health Sciences, University of Milan, Milan, Italy

**Keywords:** Oncogenes, Preclinical research

## Abstract

Standard chemotherapies for diffuse large B-cell lymphoma (DLBCL), based on the induction of exogenous DNA damage and oxidative stress, are often less effective in the presence of increased MYC and BCL-2 levels, especially in the case of double hit (DH) lymphomas harboring rearrangements of the *MYC* and *BCL-2* oncogenes, which enrich for a patient’s population characterized by refractoriness to anthracycline-based chemotherapy. Here we hypothesized that adaptive mechanisms to MYC-induced replicative and oxidative stress, consisting in DNA damage response (DDR) activation and BCL-2 overexpression, could represent the biologic basis of the poor prognosis and chemoresistance observed in *MYC/BCL-2*-positive lymphoma. We first integrated targeted gene expression profiling (T-GEP), fluorescence in situ hybridization (FISH) analysis, and characterization of replicative and oxidative stress biomarkers in two independent DLBCL cohorts. The presence of oxidative DNA damage biomarkers identified a poor prognosis double expresser (DE)-DLBCL subset, characterized by relatively higher *BCL-2* gene expression levels and enrichment for DH lymphomas. Based on these findings, we tested therapeutic strategies based on combined DDR and BCL-2 inhibition, confirming efficacy and synergistic interactions in in vitro and in vivo DH-DLBCL models. These data provide the rationale for precision-therapy strategies based on combined DDR and BCL-2 inhibition in DH or DE-DLBCL.

## Introduction

Diffuse Large B-cell lymphoma (DLBCL) is the most common non-Hodgkin lymphoma (NHL) subtype, and yet 40% of patients are resistant to current therapies [1–3], which are still based on the induction of exogenous DNA damage with anthracycline-based chemotherapy regimens representing the standard of care [1–3]. The cell of origin (COO) determined by gene expression profiling (GEP) is a well-established prognostic predictor in DLBCL [4–7]. In general, DLBCLs with a GEP signature related to activated B-cell cells (ABC subgroup) have a worse outcome compared to their germinal center B-cell (GCB) counterparts [4–7], and display higher expression levels of MYC and BCL2 [8] and oncogenic addiction to nuclear factor kappa-B (NF-kB) signaling [9]. However, despite its prognostic relevance, COO-based precision therapy has not yet translated into meaningful clinical benefits [10–12]. Besides the COO, overexpression or genomic rearrangements of the *MYC* and *BCL-2* oncogenes are powerful negative prognostic factors in DLBCL [13, 14]. Due to the fact that concurrent *MYC* and *BCL-2* rearrangements enrich for a patient’s population characterized by refractoriness to standard anthracycline-based chemotherapy, these lymphomas are now classified as a separate disease entity (HG-BCL w/DH) [15] and currently treated with more intensive chemotherapy regimens, representing a major unmet need in lymphoma therapy [16, 17]. On the other hand, the observation that a fraction of HG-BCL w/DH can be cured with standard therapies underlines the concept that the mechanisms underlying chemoresistance in MYC/BCL-2 positive DLBCL are still poorly defined. Recent evidence suggests that MYC-positive tumors are characterized by replicative and oxidative stress leading to inherent DNA damage and genomic instability [18–20]. Constitutive activation of the DNA damage response (DDR) pathway is one of the main mechanisms by which cancer cells cope with replicative stress, avoiding intolerable levels of endogenous DNA damage [21–23]. On the other hand, it is well known that BCL-2 overexpression synergizes with MYC in driving B-cell lymphomagenesis, by counteracting MYC-related proapoptotic effects and oxidative stress [24–28]. Of note, constitutive DDR activation correlates with MYC levels predicting poor prognosis [29], and therapeutic approaches targeting DDR through inhibition of the Ataxia teleangiectasia and Rad3 related (ATR)-checkpoint kinase 1/2 (CHK1/2) axis showed efficacy in preclinical models of *MYC*-positive DLBCL, including those with *TP53* mutations/deletions and *CDKN2A* loss which are mechanistically linked to anthracycline resistance [30–33].

Since chemotherapy exerts its cytotoxic effects through exogenous DNA damage and induction of reactive oxygen species (ROS), adaptive mechanisms to replicative and oxidative stress (consisting in DDR activation and upregulation of antioxidant capacity), could represent the biologic basis of the poor prognosis and chemoresistance observed in MYC/BCL-2-double expresser DLBCL and HG-BCL w/DH.

In an effort to design specific therapies for MYC/BCL-2 positive DLBCL, we first integrated targeted-GEP (T-GEP), fluorescence in situ hybridization (FISH) analysis, and functional characterization of replicative and oxidative stress biomarkers in two independent DLBCL cohorts. Since the presence of oxidative DNA damage biomarkers identified a poor prognosis DE-DLBCL subset, which was characterized by relatively higher *BCL-2* gene expression levels and enrichment in HG-BCL w/DH, we then tested therapeutic strategies based on combined DDR and BCL-2 inhibition, confirming efficacy and synergistic interactions in in vitro and in vivo HG-BCL w/DH models. These data provide the rationale for novel precision therapy strategies based on combined DDR and BCL-2 inhibition in double-hit DLBCL.

## Methods

### Patients

In the present study, we analyzed two independent patients cohorts: 69 patients from the DLCL04 study [34], a prospective randomized phase 3 clinical trial investigating the role of first-line autologous stem cell transplant (ASCT) consolidation after chemoimmunotherapy in CD20 + DLBCL, and 66 patients from a real-life cohort treated with R-CHOP/CHOP-like regimens at S. Orsola-Malpighi Hospital, Bologna (Italy), from 2007 to 2012. Patients characteristics are summarized in Table 1.

**Table 1:** Patients characteristics.

Factor	DLCL04 (*N* = 69)	Real life (*n* = 66)	*p* value	Combined (*n* = 135)
COO				
ABC	21	12	0.1	33
GCB/UNCL	48	54		102
Age	51 (18–63)	64 (17–85)	<0.01	57 (17–85)
IPI				
0–1	–	17	0.2	17
2–3	57	45		102
4–5	12	4		16
Treatment				
R-CHOP-like	39	66	–	105
R-CHOP-like + ASCT	30	–		30
MYC/BCL-2 status				
Nano				
DE	18	17	0.9	35
Non-DE	51	49		100
DH	4	3	0.9	7
γH2AX/8-OHDG				
DE	38	31	0.39	69
NON DE	31	35		66

*COO* Cell of origin, *IPI* international prognostic index, *DH* double hit, *ASCT* autologous stem cell transplant, *nano* NanoString, *DE* double expresser, *ABC* activated B-cell, *GCB/UNCL* germinal center B-cell/unclassified.

**Fig. 1:**
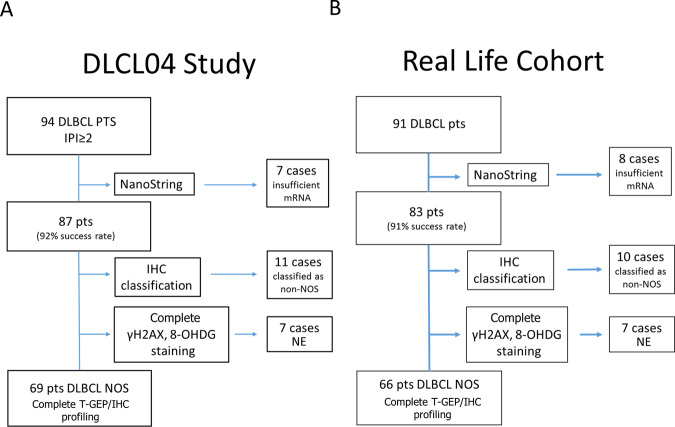
Study cohorts. **A** On the left, the discovery cohort is represented. 94 stage III-IV DLBCL patients enrolled in the DLCL04 trial with available FFPE tissue were initially considered in this analysis. T-GEP success rate was 92.6% (*n* = 87), with seven cases not yielding enough high-quality mRNA to undergo successful GEP assessment. Only cases of non-otherwise specified (NOS) histology (including those originally diagnosed as DLBCL-NOS and nowadays included in the HG-BCL provisional category) were considered; therefore 11 cases classified in different DLBCL categories were excluded. In seven cases that were not evaluable (NE), we could not retrieve enough tissue for additional immunohistochemistry studies and these cases were excluded. Sixty-nine NOS-DLBCL FFPE patient samples from the DLC04 trial were finally included in this study. **B** On the right a “real-life” validation cohort including 66 consecutive DLBCL NOS cases with available FFPE tissue for T-GEP and IHC, treated with R-CHOP/CHOP-like regimens. Success rates of T-GEP, and cases excluded for histologic classification or IHC tissue availability issues are detailed in the figure.

The study flowchart is depicted in Fig. 1 and additional details are provided in supplement.

This study was approved by the Institutional Review Boards of the participating centers, in accordance with the Declaration of Helsinki.

### Targeted gene expression profiling (T-GEP) panel

Gene expression was measured on the NanoString nCounter Analysis System (NanoString Technologies, Seattle, WA, USA). The original T-GEP panel contains 22 genes: 15 genes used to assign COO subtype [6], 5 housekeeping genes (*UBXN4, ISY1, R3HDM1, WDR55, TRIM56*); and the additional genes of interest: *MYC* and *BCL-2*. The complete list of genes, target sequences, and detailed methods are available in supplement.

### Immunohistochemistry and fluorescence in situ hybridization (FISH)

Immunohistochemistry (IHC) was centralized in Milan for the DLCL04 trial and in Bologna for the real-life control group. Primary antibody source and dilutions are shown in Table S1 and a detailed description of IHC and FISH methods is provided in supplement.

The cut-off values of 50% and 40% positive neoplastic cells were applied for BCL2 and MYC respectively [14], and 50% for γH2AX and 8-OHdG.

### Reagents, in vitro assays, and cell lines

Prexasertib was provided by the Eli Lilly company for in vitro studies, and was purchased from Selleckchem (Houston, Tx) for in vivo studies. AZD7762, MK-8776, Venetoclax and the MCL-1 inhibitor S63845 were purchased from Selleckchem (Houston, TX).

Detailed information on cell lines, 8-OHdG ELISA assay, Caspase 3/7 assay, cell cycle analyses, qPCR assays, proliferation assays (Cell Titer Glo, Promega), and western blot antibodies are provided in supplement. The Tet-OFF MYC P-4936 cell line [35, 36], which carries a conditional, tetracycline-regulated *MYC* promoter, was provided by Dr. A. Younes lab (Memorial Sloan Kettering Cancer Center, New York, NY). Inducible overexpression of BCL2 in the SUDHL5 (BCL2 negative) cell line was performed by using the Cellecta InDOXible Tet-Activated cDNA Lentiviral Expression System (custom Cellecta). Detailed methods for in vitro studies are provided in supplement.

### High-throughput screening experiments

High-throughput drug screening experiments were performed as previously described [37]. Plates were imaged after incubation with Alamar Blue on the LEADseeker^TM^ Multimodality Imaging System (GE Healthcare, Piscataway, NJ). In order to evaluate synergy, we compared the observed activity to the expected activity of the combination at that dose level under Bliss independence model [38, 39]. HTS experiments and analyses were performed at the Memorial Sloan Kettering Cancer Center, New York. Detailed information is available in supplement.

### In vivo studies

Experiments involving animals were approved by the Italian Ministry of Health and have been performed in accordance with the applicable Italian laws (D.L.vo 26/14 and following amendments), the Institutional Animal Care and Use Committee, the institutional guidelines of the European Institute of Oncology and the ARRIVE guidelines [40].

The luciferase-expressing patient-derived xenograft (PDX) line DFBL-69487-V3-mCLP [41] was obtained from the Public Repository of Xenografts (www.proxe.org). Detailed information is available in supplement.

### Statistical analysis

Survival data and correlations were analyzed retrospectively. For survival analysis, we used the Kaplan-Meier method [42] to estimate overall survival (OS). The two-tailed Student *t* test and Wilcoxon Rank test were used to estimate statistical significance. Correlations and differences in patients’ characteristics were analyzed with the chi-square and Fisher’s exact test. The PRISM software was used for the statistical analyses (v7). Significance was set at *P* < 0.05. Combination index analysis was performed using the Chou-Talalay method [43].

T-GEP statistical analyses were calculated with the R software (v3.5.0) [44]. A detailed description is provided in supplement.

## Results

### Replicative and oxidative stress biomarkers identify poor prognosis subsets of double expresser DLBCL

In order to investigate the relationship between COO classification, MYC/BCL-2 status, and replicative/oxidative stress biomarkers, we first profiled two independent case series of chemoimmunotherapy-treated DLBCL with T-GEP, FISH, and immunohistochemistry: a discovery cohort from the DLC04 study [34], and a validation cohort of patients treated in real-life clinical practice. Patients characteristics are shown in Table 1 and were similar in the two cohorts (except for median age, significantly higher in the real-life cohort), with no significant differences in the overall outcome (Figure S1). For T-GEP studies, we used a digital multiplex gene expression profiling platform (NanoString Technology) with a panel containing 22 genes (15 genes for COO subtyping according to LST algorithm [6] plus *MYC* and *BCL2* and 5 housekeeping genes). T-GEP, FISH and immunohistochemistry (IHC) profiling for c-MYC, BCL-2, the phosphorylated form of H2AX at S139 (γH2AX, a biomarker of DNA damage and DDR activation) [45, 46] and 8-hydroxy-2’-deoxyguanosine (8-OHdG, an oxidative DNA damage marker) [47] were available in 69 patients from the DLC04 study [34], and 66 patients treated in the real-life cohort (Fig. 1 A, B).

**Fig. 2:**
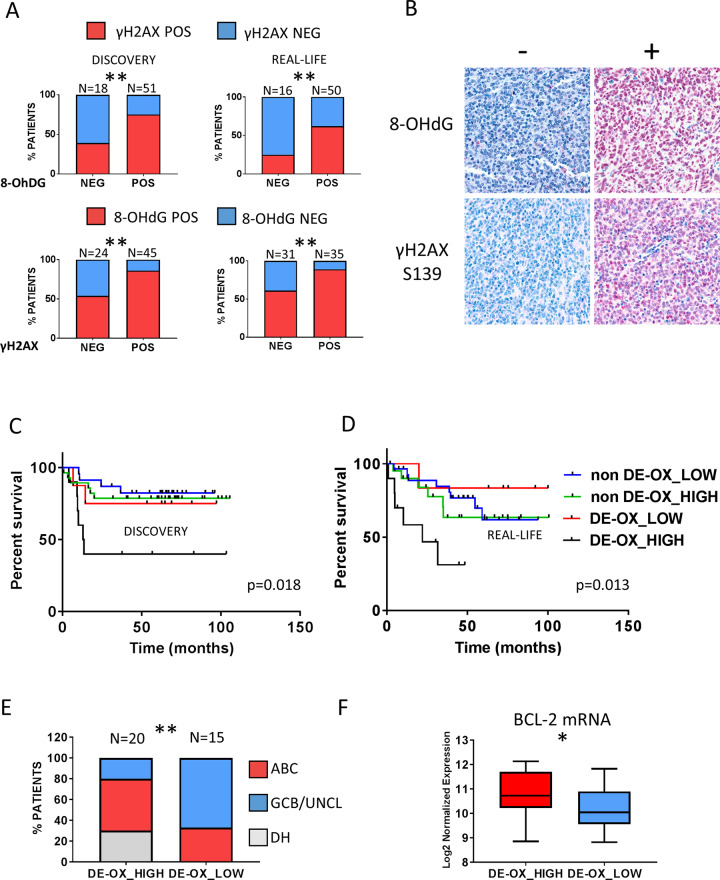
Oxidative and replicative stress biomarkers in poor prognosis DLBCL subsets. **A** In the upper panel, bar graph indicating the proportion of 8-OhDG positive and negative cases according to the expression levels of γH2AX in the discovery (left) and real-life cohort (right). In the lower panel, the proportion of γH2AX positive and negative cases according to the expression levels of 8-OhDG in the discovery (left) and real-life cohort (right). *P* value was calculated with the chi-square test. A *p* value <0.05 was considered as statistically significant: * <0.05, ** <0.01. POS (positive); NEG (negative). See also Figure S1. **B** Representative examples of 8-OHdG and γH2AX positive (right panel) and negative (left panel) DLBCLs. Original magnification, 600x. **C** OS of the discovery cohort (DLCL04; *n* = 69) according to the MYC/BCL-2 status as determined by T-GEP and to γH2AX/8-OhDG levels as assessed by IHC. DLBCL showing increased levels of *MYC* and *BCL-2* mRNA by T-GEP (*MYC/BCL-2* DE) and concurrent overexpression of γH2AX and 8-OhDG (DE-OX_high subgroup) are characterized by a worse outcome compared to all other subgroups. *P* values were calculated with the log-rank test. See also Figures S2 and S3. **D** OS of the real-life validation cohort (*n* = 66) according to the MYC/BCL-2 status as determined by T-GEP and to γH2AX/8-OhDG levels as assessed by IHC. DLBCL showing increased levels of *MYC* and *BCL-2* mRNA by T-GEP (*MYC/BCL-2* DE) and concurrent overexpression of γH2AX and 8-OhDG (DE-OX_high subgroup) are characterized by a worse outcome compared to all other subgroups. *P* values were calculated with the log rank test. See also Figure S3. **E** Bar graphs representing the proportions of different DLBCL subsets in the DE-OX_high and DE-OX_low subgroups (analysis of the whole cohort: discovery + validation), showing relative increase in ABC cases and significant enrichment HG-BCL w/DH in the DE-OX_high subgroup. See also Table 2 for detailed statistics. See also Figure S3. **F** Box plot graph showing *BCL-2* mRNA levels as assessed by T-GEP in the DE-OX_high vs DE-OX_low subgroup. Differences between groups were calculated with the Student *T* test. *p < 0.05, **p < 0.01.

The two cohorts displayed similar patterns of γH2AX and 8-OHdG expression (cut-off value 50% of positive cells, with 55% and 47% of cases showing dual nuclear positivity for γH2AX and 8-OHdG in the DLCL04 and real-life cohort respectively). The expression levels of γH2AX and 8-OHdG were significantly correlated, with the 8-OHdG positive subgroup showing a significantly higher fraction of γH2AX positive cases, as compared to the 8-OHdG negative subset. In line with this observation, the proportion of 8-OhDG-positive samples was significantly increased in the γH2AX-positive subset in both cohorts (Fig. 2 A,B).

In order to define the prognostic implications of γH2AX and 8-OHdG expression with respect to the COO and MYC/BCL-2 status, we analyzed the impact of these variables on OS rates in the two cohorts.

Although there was a significant correlation between *MYC* and *BCL-2* mRNA (assessed with T-GEP) and protein levels (assessed by IHC) (Figure S2A), MYC/BCL-2 status evaluated by T-GEP outperformed IHC for prognostic stratification in both cohorts (Table S2 and Figure S2B) (patients were classified as high and low *MYC* or *BCL-2* expressers based on the median normalized *MYC* and *BCL-2* mRNA levels in the respective cohorts).

In both patients cohorts, dual nuclear positivity for γH2AX and 8-OHdG identified a *MYC/BCL-2* mRNA Double Expresser (DE)-DLBCL subset characterized by dismal outcome (hereafter defined as DE-OX_high) (Fig. 2 C, D). Interestingly *MYC/BCL-2* mRNA DE cases with low oxidative DNA damage (DE-OX_low) had a very favorable outcome, with OS rates comparable to non-DE cases (Fig. 2 C, D). Similar trends were observed in both cohorts (Fig. 2 C, D) and a cumulative analysis (*n* = 135 patients) confirmed a significantly worse OS rate for the DE-OX_high subgroup, compared to all other subgroups (Figure S3A).

To further define the biologic characteristics of the DE-OX_high subgroup we investigated COO subtyping and the presence of *MYC* and *BCL-2* rearrangements (HG-BCL w/DH) across different patients’ subsets (DE-OX_high vs DE-OX_low). Interestingly we found that the DE-OX_high subgroups were enriched in HG-BCLs w/DH (Fig. 2E), showed a relative increase in ABC cases, and were characterized by relatively higher *BCL-2* mRNA levels compared to the DE-OX_low subsets (Fig. 2F). Notably, all but one HG-BCL w/DH clustered in the DE-OX_high subgroups (Table 2). The outcome of DE-OX_HIGH subset was dismal irrespective of the DH status, with similarly poor OS rates observed in DH and non-DH cases (Figure S3B). These data indicate that the expression of replicative and oxidative stress biomarkers such as γH2AX and 8-OHdG could define a subset of *MYC/BCL-2* DE DLBCL with specific molecular features and characterized by a worse outcome.

**Table 2:** Characteristics of the DE-OX_low and DE-OX-high subgroups (analysis of the whole cohort: discovery + validation).

	DE-OX_low (*n* = 15)	DE-OX_high (*n* = 20)	*p* value
Treatment			
R-CHOP_like	10	13	0.99
R-CHOP_like + ASCT	5	7	
COO			
GCB	5	6	0.99
UNCL	5	4	0.45
ABC	5	10	0.49
DH^a^	0	6	0.027
DH + ABC	5	16	0.013

*COO* cell of origin, *DH* double hit, *ASCT* autologous stem cell transplant, *DE* double expresser, *ABC* activated B-cell, *GCB/UNCL* germinal center B-cell/unclassified.

*P* value was calculated with the Fisher’s exact test.

^a^The remaining 1 DH case clustered in the non-DE subset. All DH cases were of GCB origin.

### Functional characterization of replicative and oxidative stress biomarkers following DDR inhibition in DLBCL

**Fig. 3:**
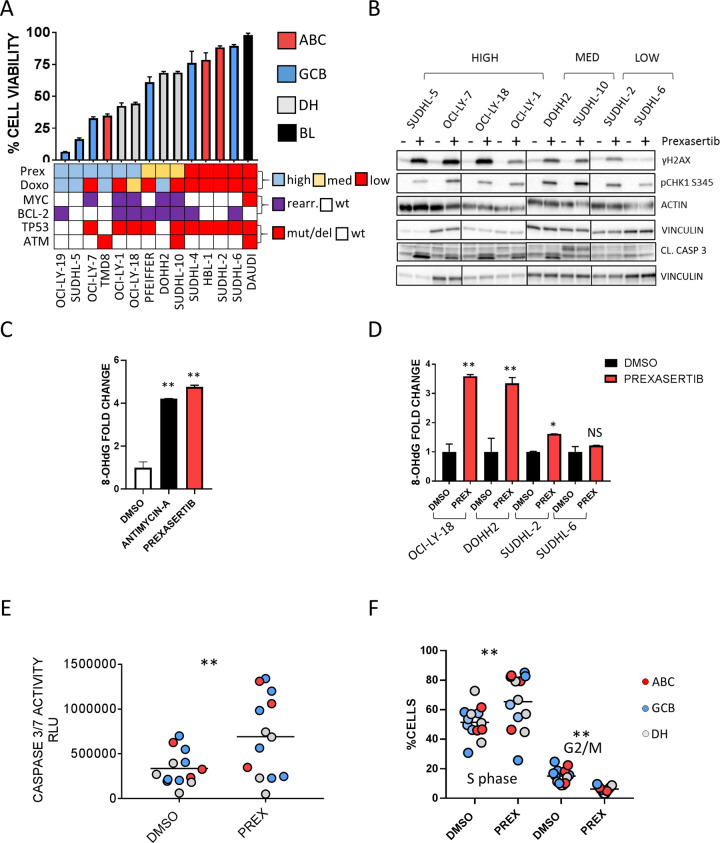
DDR inhibition with Prexasertib determines oxidative DNA damage accumulation and apoptosis in DLBCL cell lines. **A** Bar graph showing percentages of cell viability measured with the Cell Titer Glo assay (Promega) in 13 DLBCL cell lines and 1 BL cell line (Daudi) treated with Prexasertib 125 nM for 24 h. Below the graph, a heat map showing: 1- information on Prexasertib (Prex) and Doxorubicin (Doxo) sensitivity defined as high >50% inhibition; med 25–50% inhibition; low <25% inhibition. 2- annotation regarding *MYC, BCL-2, TP53, ATM* genomic alteration status in all cell lines (rearr.: rearrangement; wt: wild type; mut/del: mutation/deletion). Detailed reference of genomic data annotation is available in supplement. Error bars represent the standard error of the mean (SEM) of triplicate experiments. See also Figure S4. **B** Representative western blot assays depicting the effects of 125 nM Prexasertib on expression levels of γH2AX, pCHK1 S345, Caspase 3 cleavage after 6 h of incubation in DLBCL cell lines characterized by high, intermediate (med), and low sensitivity to Prexasertib. **C** Bar graph showing 8-OhDG levels in DNA extracted from DOHH2 cells treated with DMSO or Prexasertib (250 nM) and Antimycin A (100 nM) for 6 h. Error bars represent standard error of the mean (SEM) of triplicate experiments. Differences between groups were calculated with the Student *T* test. *p < 0.05, **p < 0.01. **D** Bar graph showing 8-OhDG levels in DNA extracted from OCI-Ly-18, DOHH2, SUDHL-2, and SUDHL-6 cells treated with DMSO or Prexasertib (125 nM) (PREX) for 6 h. Error bars represent standard error of the mean (SEM) of triplicate experiments. Differences between groups were calculated with the Student *T* test. *p < 0.05, **p < 0.01. See also Figure S4. **E** Scatter plot representing the effects of 125 nM Prexasertib (PREX) on apoptosis in DLBCL cell lines. Cells were incubated with Prexasertib for 24 h and caspase 3/7 activation assessed with the Caspase Glo 3/7 assay system (Promega™). Each point represents the mean of three independent experiments. *P* value was calculated with the paired Student’s *T* test. See also Figure S4 and S5. RLU (relative light units). **F** Scatter plot representing the effects of 125 nM Prexasertib (PREX) on cell cycle in DLBCL cell lines, showing significant accumulation of DLBCL cells in the S phase and reduction of the G2/M phase. Cells were incubated with Prexasertib for 24 h and changes in cell cycle fractions were assessed by flow cytometry with propidium iodide staining. Each point represents the mean of three independent experiments. *P* value was calculated with the paired Student’s *T* test. See also Figure S5.

To further investigate these findings we treated a panel of DLBCL cell lines with the CHK1/2 inhibitor Prexasertib [48], and assessed the effects of treatment on γH2AX and 8-OHdG levels. Prexasertib showed antiproliferative activity at sub-micromolar concentrations across multiple cell lines, irrespective of the cell doubling time, COO, *MYC/BCL-2* rearrangements, *TP53,* and (Ataxia Telangiectasia Mutated) *ATM* status (Fig. 3A and Figure S4A,B). As opposite, the in vitro efficacy of the DNA-damaging agent Doxorubicin was closely related to the *TP53* wild-type status (Figure S4C). As previously reported [48, 49], treatment with Prexasertib resulted in reduced clearance of DNA damage foci and consequent upstream DDR activation as demonstrated by increased γH2AX and p-CHK1 S345 levels respectively (Fig. 3B). While in some cell lines Prexasertib and Doxorubicin had similar effects on cell viability and DNA damage accumulation, DDR inhibition by Prexasertib significantly increased γH2AX levels in cell lines where Doxorubicin failed to determine significant DNA damage (Figure S4D). Furthermore, Prexasertib-induced DDR inhibition resulted in increased levels of DNA oxidation as assessed with a 8-OHdG ELISA assay (Fig. 3C,D). The extent of 8-OHdG induction was similar to that observed after treatment with known oxidative stress-inducing agents (Fig. 3C), such as Antimycin A [50], and was more prominent in Prexasertib-sensitive cell lines (Fig. 3D), showing a dose-dependent pattern (Figure S4E). Prexasertib treatment induced significant apoptosis in DLBCL cell lines irrespective of *TP53* status and was associated with significant accumulation of cells in S phase of the cell cycle, in line with previous observations [51] (Fig. 3E, F and Figures S4F and S5A-F). These data, together with our observations on γH2AX and 8-OHdG expression patterns in DLBCL tissues, suggest that oxidative stress could be a major source of inherent DNA damage contributing to constitutive DDR activation in DLBCL, and indicate that DDR inhibition induces oxidative DNA damage accumulation, cell cycle arrest and apoptosis in DLBCL cell lines.

### BCL-2 inhibition enhances the activity of DDR inhibitors in in vitro DLBCL models

**Fig. 4:**
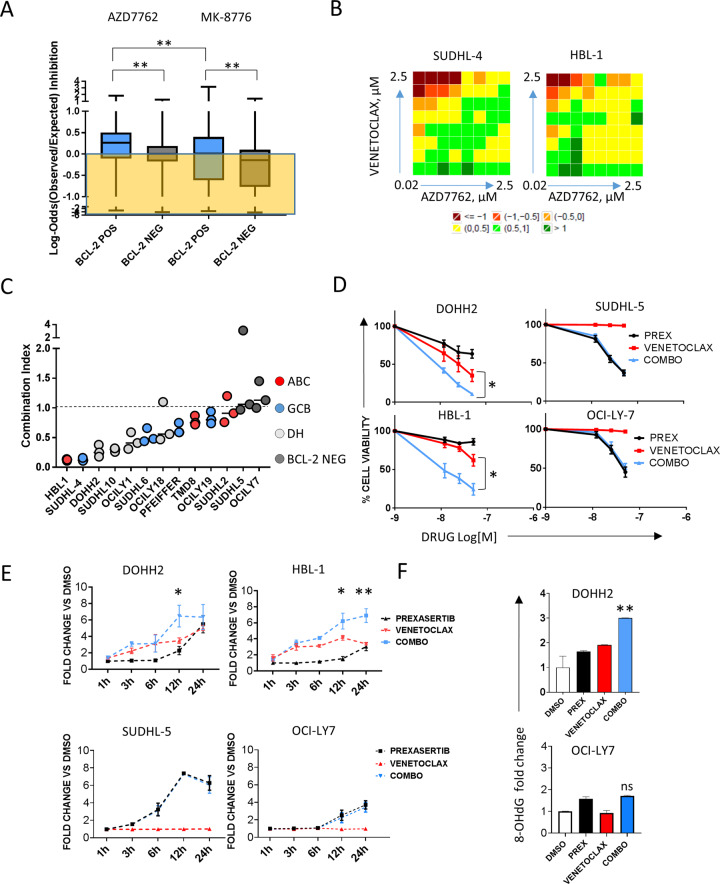
BCL-2 blockade enhances antilymphoma activity of DDR inhibitors in vitro. **A** High throughput screening of the checkpoint kinase inhibitors AZD7762 or MK-8776 combined with the BCL-2 inhibitor Venetoclax (ABT-199) in 10 DLBCL cell lines. Box plot graphs summarize the results of the combinatorial drug screening analyzed with the Bliss model in 10 DLBCL cell lines treated for 72 h with the indicated drug combinations. The *Y* axis indicates the ratio between observed and expected inhibition in a log scale. Values above the “0” line indicate enhanced antiproliferative effects for the combinations. See HTS statistical analysis in the methods section and supplemental data for detailed information. See also Figure S6. **B** Representative examples of combination experiments of AZD7762 plus Venetoclax in HBL-1 and SUDHL-4 cells. Combination responses are examined using a 8 × 8 viability matrix representing the ratio between observed/expected inhibition in log scale, which was measured after 72 h post treatment. Full data are shown in supplement. **C** High throughput screening results were confirmed by independent experiments with the CHK1/2 inhibitor Prexasertib and Venetoclax, using the Cell Titer Glo assay in 13 DLBCL cell lines, and results were calculated using Combination Index analysis. Three doses of Prexasertib (12.5, 25, 50 nM) were combined with three doses of Venetoclax (12.5, 25, 50 nM) for 24 h, allowing three combinatory values for each cell line. Values below 1 indicate synergistic interactions. ABC cell lines overexpressing BCL-2 are depicted in red, GCB in blue, DH in light gray, BCL-2 negative in dark gray. See also Figures S7 and S8. **D** Representative examples of Cell Titer Glo experiments in two BCL-2 positive (DOHH2, HBL-1), and 2 BCL-2 negative (OCILY-7, SUDHL-5) cell lines. Cells were incubated with increasing concentrations of Prexasertib (PREX) and Venetoclax (12.5, 25, 50 nM), and cell viability was assessed after 24 h. Error bars represent standard error of the mean (S.E.M) of triplicate experiments. Differences between groups were calculated with the Student *T* test. *p < 0.05, **p < 0.01. See also Figures S7, S8 and S9. **E** Graph showing fold changes over time in caspase 3/7 activity in BCL-2 positive (DOHH2, HBL-1) vs BCL-2 negative (OCILY-7, SUDHL-5) cell lines treated with Prexasertib, Venetoclax, or the combination. Cells were treated with 50 nM of Prexasertib, Venetoclax and the combination for the indicated time. Error bars represent standard error of the mean (S.E.M) of triplicate experiments. Differences between groups (combo vs Venetoclax and Prexasertib as single agents) were calculated with the Student *T* test. *p < 0.05, **p < 0.01. **F** Bar graph showing early changes in DNA oxidation levels in DOHH2 and OCILY-7 cells treated with Prexasertib, Venetoclax or the combination for 1 h. After treatment, DNA was extracted and 8-OHdG levels were assessed with a dedicated ELISA assay. The combination determined synergistic induction of 8-OHdG in the BCL-2 positive DOHH2 cells and was ineffective in the BCL-2 negative cell line OCILY-7. Error bars represent standard error of the mean (S.E.M) of triplicate experiments. Differences between groups were calculated with the Student *T* test. *p < 0.05, **p < 0.01.

Since constitutive expression of replicative and oxidative stress biomarkers defined a subset of DE-DLBCL characterized by worse outcome and increased *BCL-2* gene expression levels, we next investigated the therapeutic implications of these findings in in vitro DLBCL models, by combining different DDR inhibitors with the selective BCL-2 inhibitor Venetoclax. In a preliminary experiment, using a combinatorial high-throughput drug screening (HTS) approach we first combined two DDR inhibitors (the CHK1/CHK2 inhibitor AZD-7762 and the selective CHK1 inhibitor MK-8776) with the BCL-2 inhibitor Venetoclax in 10 DLBCL cell lines. As shown in Fig. 4A, BCL-2 inhibition significantly enhanced the antiproliferative activity of CHK inhibitors in BCL-2 positive cell lines (ABC-derived or GCB-derived harboring *BCL-2* rearrangements, Figure S6 A-D). In general, better trends were observed with CHK1/2 inhibition (AZD-7762) than with selective CHK1 inhibition (MK-8776) (Fig. 4A). BCL-2 protein expression data and HTS results of individual cell lines are shown in supplement (Figure S6 A-E). Individual matrices of 1 representative GCB cell line with *BCL-2* translocation (SUDHL-4) and 1 ABC-derived cell line (HBL-1) treated with AZD7762 are represented in Fig. 4B (complete data are shown in HTS data supplement). Since we described a significant enrichment of HG-BCL w/DH cases in the DE-Ox high subset, we then extended our cell line panel including different DH DLBCL models, and validated HTS results by cell-titer-glo assay using the CHK1/2 inhibitor Prexasertib. As observed with AZD-7762, BCL-2 inhibition significantly enhanced the efficacy of Prexasertib in multiple DLBCL cell lines (Fig. 4C,D and Figure S7). In line with our HTS data, the most favorable interactions were observed in BCL-2 positive DLBCL cell lines, including different *BCL-2* rearranged and DH lymphoma models (Fig. 4 C and D). The observation that Prexasertib-induced apoptosis was less pronounced in *BCL-2*-rearranged and DH cell lines (Figure S5 C,D) further corroborates these findings. BCL-2 protein expression data in this validation panel are shown in supplement (Figure S8A). Although the efficacy of Prexasertib in combination with Venetoclax in DE cell lines was not affected by baseline γH2AX and 8-OHdG levels, cell lines with low levels of γH2AX and 8-OHdG showed a trend toward a decreased sensitivity to single-agent Venetoclax (Figure S8B-G). Combinatory treatment with Prexasertib and Venetoclax synergistically induced apoptosis as demonstrated by time-dependent increase in caspase 3/7 cleavage in BCL-2 positive ABC and DH lymphoma cell lines (Fig. 4E). In line with the data shown in Fig. 2 and with the role of BCL-2 in regulating the oxidative stress response, these changes were associated with early induction of oxidative DNA damage (Fig. 4F). Taken together these data indicate that selective BCL-2 blockade enhances the in vitro activity of CHK inhibitors in multiple BCL-2 positive DLBCL models including HG-BCL w/DH, by inducing oxidative DNA damage and apoptosis. Of note, single-agent Prexasertib or oxidative stress-inducing agents such as doxorubicin or H2O2 did not determine significant BCL-2 induction in either BCL-2 negative or BCL-2 positive cell lines (Figure S9A). Finally, the MCL-1 inhibitor S68345 enhanced Prexasertib efficacy in BCL-2 negative cell lines (Figure S9B), suggesting that dual blockade of the DDR and alternative BCL-2 family members could be of value in DLBCLs with low BCL-2 expression.

### Differential role of *MYC* and *BCL-2* in modulating the efficacy of checkpoint kinase inhibition

**Fig. 5:**
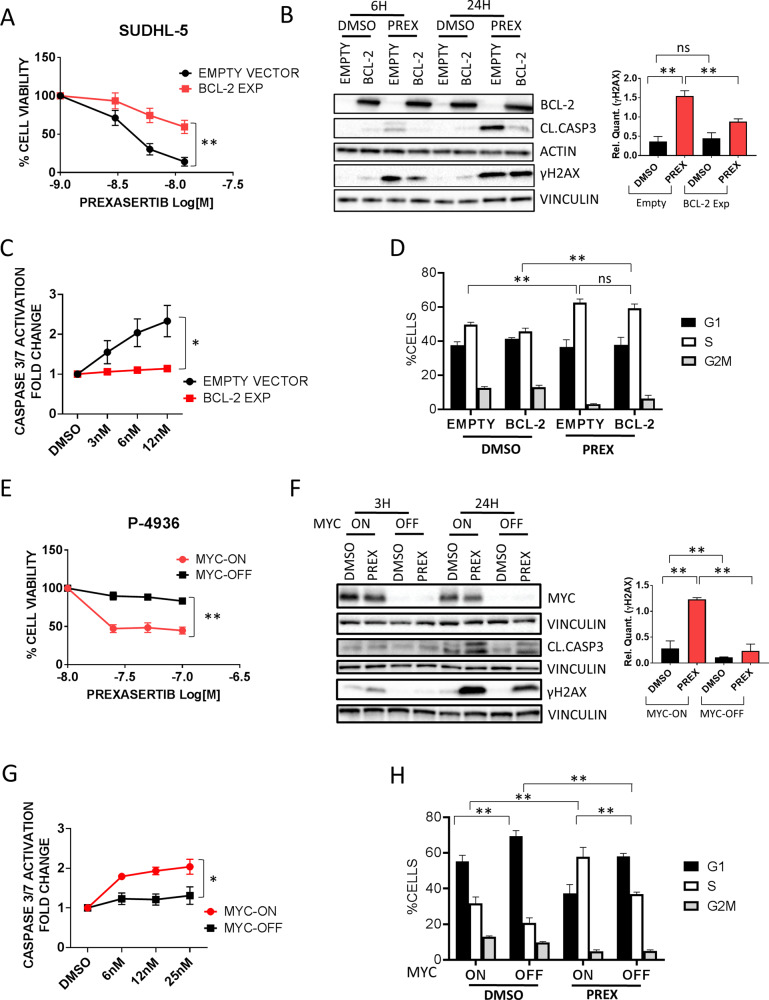
Role of BCL-2 and MYC in modulating sensitivity to DDR inhibition. **A** Cell Titer Glo assay showing the effects of 3 doses of Prexasertib (3, 6, and 12 nM) for 24 h in the absence (EMPTY VECTOR) or in the presence of BCL-2 (BCL-2 EXP). SUDHL-5 cells (transfected with EMPTY VECTOR or a BCL-2 TET-ON inducible system (BCL-2 EXP) were preincubated with doxycycline 1 μg/ml for 24 h and then treated with Prexasertib at the indicated doses (see also Figure S10A). Error bars represent standard error of the mean (S.E.M) of triplicate experiments. Differences between groups were calculated with the Student *T* test. *p < 0.05, **p < 0.01. **B** On the left, representative western blot assay showing the effects of Prexasertib (6 nM) (PREX) on γH2AX induction and caspase 3 cleavage (CL. CASP 3) in the presence or absence of BCL-2, after 6 and 24 h of incubation. On the right, quantitative densitometry analyses (ImageJ software, western blots are shown in Figure S10B) showing normalized γH2AX levels vs vinculin after 6 h of incubation with Prexasertib in the presence or absence of BCL-2: γH2AX induction by Prexasertib was significantly attenuated in the presence of BCL-2. Error bars represent standard error of the mean (S.E.M) of triplicate experiments. Differences between groups were calculated with the Student *T* test. *p < 0.05, **p < 0.01. **C** Graph showing fold changes in caspase 3/7 activity in SUDHL-5 cells treated with increasing doses of Prexasertib (3,6,12 nM) for 12 h in the presence or absence of BCL-2 (BCL-2 EXP vs EMPTY VECTOR), showing significant inhibition of caspase 3/7 cleavage in BCL-2 overexpressing SUDHL-5 cells. Error bars represent standard error of the mean (S.E.M) of triplicate experiments. Differences between groups were calculated with the Student *T* test. *p < 0.05, **p < 0.01. **D** Bar graph showing the effects of DMSO or Prexasertib (PREX) on cell cycle phases in SUDHL-5 cells in the presence or absence of BCL-2. After overexpression of BCL-2 (BCL-2) or the empty vector (EMPTY) for 24 h, cells were incubated with 12 nM Prexasertib for additional 24 h and cell cycle phases assessed by flow cytometry (propidium iodide staining). Error bars represent standard error of the mean (S.E.M) of triplicate experiments. Differences between groups were calculated with the Student *T* test. *p < 0.05, **p < 0.01. **E** Cell Titer Glo assay showing the effects of three doses of Prexasertib (25, 50, and 100 nM) for 24 h in the absence of MYC (MYC OFF) or in the presence of MYC (MYC-ON) in P-4936 cells. P-4936 cells (carrying a tetracycline inducible promoter, TET-OFF MYC) were preincubated with doxycycline 1 μg/ml for 6 h to downregulate MYC, and then treated with Prexasertib at the indicated doses (See also Figure S11A). Error bars represent standard error of the mean (S.E.M) of triplicate experiments. Differences between groups were calculated with the Student *T* test. *p < 0.05, **p < 0.01. **F** Representative western blot assay showing the effects of Prexasertib (25 nM for 3 and 24 h of incubation) (PREX) on γH2AX induction and caspase 3 cleavage in the presence (MYC-ON) or absence of MYC (MYC-OFF). On the right, quantitative densitometry analyses (ImageJ software, western blots are shown in Figure S11B) showing normalized γH2AX levels vs vinculin after 3 h of incubation with Prexasertib in MYC-ON and MYC-OFF P-4936 cells. γH2AX induction by Prexasertib was significantly attenuated in the absence of MYC. Error bars represent standard error of the mean (S.E.M) of triplicate experiments. Differences between groups were calculated with the Student *T* test. *p < 0.05, **p < 0.01. **G** Graph showing fold changes in caspase 3/7 activity in P-4936 cells treated with increasing doses of Prexasertib (6, 12, 25 nM) for 12 h in the presence or absence of MYC (MYC-ON vs MYC-OFF), showing significant inhibition of caspase 3/7 cleavage in MYC-OFF P-4936 cells. Error bars represent standard error of the mean (S.E.M) of triplicate experiments. Differences between groups were calculated with the Student *T* test. *p < 0.05, **p < 0.01. **H** Bar graph showing the effects of DMSO or Prexasertib (PREX) on cell cycle phases in P-4936 cells in the presence or absence of MYC. After preincubation with doxycycline (MYC-OFF) or DMSO (MYC-ON) for 24 h, cells were incubated with 25 nM Prexasertib for additional 24 h and cell cycle phases assessed by flow cytometry (propidium iodide staining). Error bars represent standard error of the mean (S.E.M) of triplicate experiments. Differences between groups were calculated with the Student *T* test. *p < 0.05, **p < 0.01.

To determine the effects of BCL-2 overexpression on the therapeutic efficacy of DDR inhibition, we generated a Tet-on inducible system to overexpress BCL-2 in the Prexasertib-sensitive BCL-2 negative cell line SUDHL-5. Cells were preincubated with doxycycline 1 μg/ml for 24 h to overexpress BCL-2, and then were treated with DMSO or Prexasertib for 24 h (Figure S10A). BCL-2 overexpression, while not exerting significant effects on cell proliferation in untreated cells, significantly decreased the efficacy of Prexasertib in this system, indicating that BCL-2 promotes resistance to DDR inhibition (Fig. 5A and S10A). These changes were associated with an attenuated induction of γH2AX and with a decreased caspase 3 cleavage in cells overexpressing BCL-2 (Fig. 5B,C and S10A,B). BCL-2 overexpression did not determine significant changes in cell cycle dynamics under Prexasertib treatment (Fig. 5D).

To assess the role of MYC in regulating the antiproliferative activity of Prexasertib, we used the P-4936 cell line, which carries a conditional, tetracycline-regulated (Tet-OFF) *MYC* promoter [35, 36]. Cells were pre-treated with DMSO or with doxycycline for 6 h to abrogate MYC expression, and then incubated with Prexasertib for 24 h (Fig. 5E-H and S11A-C). MYC silencing with doxycycline significantly reduced cell proliferation and baseline γH2AX expression (Figure S11A-C). Importantly MYC depletion reduced the antiproliferative effects of Prexasertib in these cells, which was associated with impaired γH2AX induction, decreased caspase 3 cleavage, and attenuated effects on cell cycle dynamics (Fig. 5E–H). These data suggest that MYC and BCL-2 may modulate the sensitivity to CHK1 inhibition in opposite ways: in fact, while high MYC expression could be associated with increased DDR activation and enhanced susceptibility to DDRi-induced DNA damage, overexpression of BCL-2 may significantly decrease the therapeutic activity of DDR inhibitors, providing mechanistic rationale of dual blockade of DDR and BCL-2 in DE and DH lymphomas.

### BCL-2 inhibition enhances the activity of DDR inhibitors in vivo in DH PDX models

**Fig. 6:**
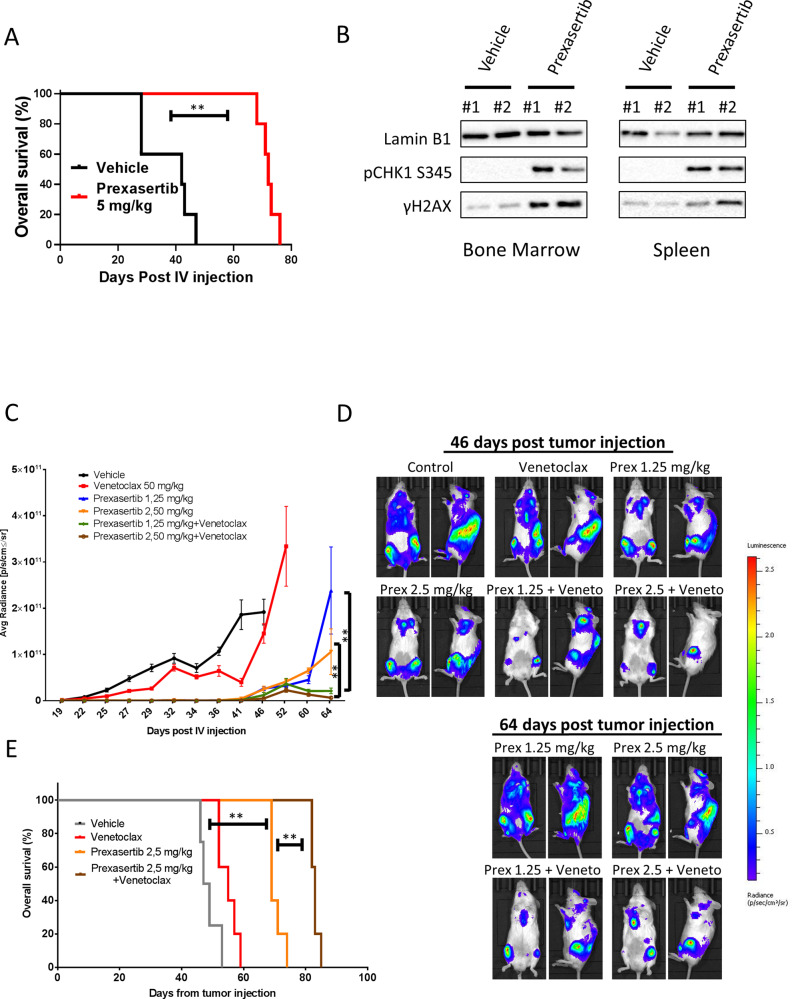
Prexasertib plus Venetoclax extends survival in vivo in a PDX DH lymphoma model. **A** Overall survival curve of PDX mice treated with vehicle (*n* = 5) or two different doses of Prexasertib (5 and 10 mg/kg BID 3 times/week) (*n* = 5 in each dose level). Cells (10^6^) were xenografted via tail vein injection into six- to eight-week-old female NSG mice (Charles River, Italy). Tumor growth was monitored three times per week by whole-body imaging on an IVIS Lumina III platform. Mice were treated for three weeks (nine doses). *P* values were calculated with the log rank test. **B** Western blot showing the in vivo effects of Prexasertib (5 mg/kg BID three times/week) on γH2AX and p-CHK1-S345 levels. Mice were sacrificed at the end of treatment (6 h after last dosing), and tissues were harvested immediately after sacrifice. **C** Combination experiment of Prexasertib and Venetoclax in the DH lymphoma PDX model (DFBL-69487-V3-mCLP). NSG mice were treated with vehicle (*n* = 5), Prexasertib (1.25 and 2.5 mg/kg BID three times/week) (*n* = 5 in each dose level), Venetoclax (50 mg/Kg/daily (five days/week) by oral gavage (*n* = 5) and the combinations (*n* = 5 each). Mice were treated for three weeks. Differences between groups were calculated with the Student *T* test. *p < 0.05, **p < 0.01. **D** Representative IVIS imaging of mice treated with vehicle (control), Venetoclax, Prexasertib (Prex) 1.25 mg/kg, Prexasertib 2.5 mg/kg, and the combinations at the indicated time points. All vehicle and Venetoclax-treated mice were sacrificed before day 64. **E** Overall survival curve of PDX mice treated with vehicle, two different doses of Prexasertib (1.25 and 2.5 mg/kg three times/week) (*n* = 5 in each dose level), Venetoclax 50 mg/Kg (five days/ week) (*n* = 5) and the combinations (*n* = 5 each). Mice were treated for a total of 21 days. *P* values were calculated with the log rank test. See also Figure S12.

To assess whether our in vitro results could be confirmed in in vivo lymphoma models, we used a PDX mouse model harboring a double *MYC* and *BCL-2* rearrangement and a 17p deletion (*TP53* loss) [42]. Treatment with Prexasertib as single-agent significantly extended the survival of mice bearing DH lymphomas (Fig. 6A). Of note, a short course 3-week therapy schedule was sufficient to extend survival of several weeks in this mouse model. In order to assess the in vivo effects of CHK inhibition, lysates from bone marrow and spleens harvested after 6 h of vehicle or Prexasertib administration were subjected to western blotting. In line with our in vitro data, Prexasertib treatment resulted in increased γH2AX and p-CHK1 s345 levels, which are established biomarkers of DDR inhibition, indicative of DNA damage accumulation and ATR-dependent CHK1 phosphorylation (Fig. 6B). In an independent experiment, we investigated the efficacy of Prexasertib in combination with the BCL-2 inhibitor Venetoclax. In vivo combination therapy with Prexasertib and Venetoclax exerted synergistic effects in our DH PDX model. Prexasertib as single-agent confirmed high antitumor activity resulting in extended survival. On the contrary, Venetoclax alone had no substantial antitumor activity. However, the combination of Prexasertib and Venetoclax resulted in enhanced tumor growth inhibition and prolonged survival, as compared to either drug administered as single agent (Fig. 6C–E). Interestingly these synergistic effects were observed after only one cycle (3 weeks) of combined treatment. We did not observe significant weight loss in mice treated with Prexasertib, Venetoclax, or the combination (Figure S12 A and B). Collectively these data suggest that combined DDR and BCL-2 inhibition could be an effective treatment strategy in DH lymphoma models, including those with defective p53 axis.

## Discussion

In an effort to understand the functional basis of the intrinsic chemoresistance associated with increased MYC and BCL-2 levels in a significant fraction of DLBCL, we hypothesized that: 1) Overexpression of DDR and oxidative DNA damage markers (γH2AX and 8-OHdG) could identify poor prognosis DLBCL subsets. 2) Pharmacologic inhibition of the DDR and antioxidant response through combined CHK1/2 and BCL-2 blockade could unleash endogenous replicative and oxidative stress resulting in synergistic therapeutic activity in MYC/BCL-2 positive DLBCL.

To address these hypotheses, we first profiled two independent DLBCL cohorts with T-GEP, FISH, and IHC, in order to define COO subtyping, MYC, and BCL-2 status and expression levels of biomarkers of DDR activation and oxidative DNA damage (γH2AX and 8-OHdG). We demonstrated that: 1) expression levels of the DNA damage marker γH2AX and the oxidative DNA damage marker 8-OHdG are tightly associated, suggesting that oxidative stress could be a major source of inherent DNA damage contributing to constitutive DDR activation in DLBCL (Fig. 2); 2) Dual positivity for γH2AX and 8-OHdG is significantly associated with adverse outcome in the MYC/BCL-2 positive DLBCL subset (Fig. 2); 3) *MYC/BCL-2* mRNA DE DLBCL overexpressing γH2AX and 8-OHdG (DE-OX high) are enriched of ABC and DH cases, and are characterized by increased *BCL-2* mRNA expression compared to their γH2AX and 8-OHdG negative DE counterparts. Importantly all but one HG-BCL w/DH cases clustered in the DE-OX high subgroup (Fig. 2, Table 2). These data indicate that a subgroup of ABC DLBCL and HG-BCL w/DH are characterized by high levels of inherent oxidative DNA damage, these features being associated with increased *BCL-2* expression levels. Given the poor prognosis of these DLBCL subsets, the well-established oncogenic cooperation between MYC and BCL-2, and the known role of BCL-2 in oxidative stress response, these data are in line with a model whereby BCL-2 overexpression and constitutive DDR activation could provide a tolerance mechanism to MYC-induced replicative and oxidative stress. Since antracyclines exert their cytotoxic activity at least in part by increasing ROS levels [52, 53] and determining oxidative DNA damage, lymphoma subsets displaying inherent tolerance to oxidative DNA damage through constitutive DDR activation and BCL-2 overexpression could be intrinsically resistant to current antracycline-based chemotherapeutic regimens. The results of our in vitro experiments support this hypothesis since DDR inhibition by Prexasertib determined oxidative DNA damage accumulation (Fig. 3), which was further enhanced by the addition of Venetoclax (Fig. 4). Notably, treatment with ROS-inducing agents (Antimycin A) and Prexasertib determined accumulation of oxidative DNA damage to similar extents, suggesting that constitutive DDR activation could have a major role in preventing intolerable levels of DNA damage and genomic instability induced by endogenous oxidative stress (Fig. 3). BCL-2 blockade with Venetoclax enhanced the antilymphoma activity of checkpoint kinase inhibitors in multiple BCL-2 positive cell lines (including ABC and DH DLBCL models) resulting in increased apoptosis (Fig. 4). While enforced BCL-2 expression significantly decreased the efficacy of single-agent DDR inhibition by attenuating DNA damage accumulation and apoptosis induction (Fig. 5), on the contrary, MYC overexpression was associated with increased sensitivity to DDR inhibitors, and enhanced apoptotic response in line with previous reports [21–23]. Interestingly, while BCL-2 overexpression did not exert significant effects on cell proliferation, ectopic MYC expression was associated with increased cell proliferation and enhanced γH2AX expression, indicative of increased replicative stress and DDR activation (Figure S11). These observations underline the intrinsic correlation between MYC and the DDR, and the importance of dual targeting of the DDR and BCL-2 in MYC/BCL-2 positive lymphoma in order to maximize the therapeutic efficacy. These data were confirmed in vivo, in a double hit PDX model with *TP53* loss (Fig. 6). Interestingly single-agent Venetoclax had negligible antilymphoma activity in vivo in line with data from early phase clinical trials in DLBCL [54]. The recent demonstration of synergy between Venetoclax and Tygecycline in DH lymphoma models is in line with our findings, indicating that therapeutic strategies based on synthetic lethal targeting of oxidative stress could be of value in DH-DLBCL [55].

In summary, these data indicate that increased tolerance to replicative and oxidative stress through DDR activation and BCL-2 overexpression could be a unifying feature of poor prognosis MYC positive DLBCL subsets such as ABC and HG-BCL w/DH, which could be the basis for a tailored therapeutic approach. In this light, novel therapies based on dual targeting of DDR and antioxidant response could determine significant improvements in DLBCL therapy. This strategy, based on unleashing endogenous MYC-related replicative and oxidative stress rather than inducing exogenous DNA damage, represents a significant innovation, which could provide less toxic alternatives to conventional chemotherapy.

## Supplementary information

Supplementary material
