# Supplementary material for: Dual targeting of the DNA damage response pathway and BCL-2 in diffuse large B-cell lymphoma
Source: Leukemia. 2021 Jul 24;36(1):197–209. doi: 10.1038/s41375-021-01347-6 (PMC8727301; doi:10.1038/s41375-021-01347-6)
Supplement: Supplementary file 1 — Supplementary material [file 41375_2021_1347_MOESM1_ESM.pdf]

SUPPLEMENTAL INFORMATION

Supplemental methods

Patient's characteristics

399 patients with high risk DLBCL [age-adjusted international prognostic index score (aaIPI) 2-3] were originally enrolled in the DLCL04 trial [1]. Median follow-up was 72 months. The results of this study did not support the role of first line intensification in DLBCL as 5-year overall survival (OS) rates were similar in the no transplantation group (77%) vs the transplantation group (78%). The overall outcome of the patients analyzed in the DLCL04 cohort of the present study was superimposable to the outcome of the original study (1) (5-year OS 74% vs 78% p=ns) (Figure S1).Only cases originally diagnosed as non-otherwise specified (NOS) DLBCL, (including those originally diagnosed as DLBCL-NOS and nowadays included in the HG-BCL provisional category) [2] were considered. 94 stage III-IV DLBCL patients enrolled in the DLCL04 trial with available FFPE tissue were initially considered in this analysis (discovery cohort). T-GEP success rate was 92.6% (n=87), with 7 cases not yelding enough high-quality mRNA to undergo successful GEP assessment. Only cases of non-otherwise specified (NOS) histology were considered. Therefore 11 cases classified in different DLBCL categories were excluded. In 7 cases we could not retrieve enough tissue for additional immunohistochemistry studies and these cases were excluded. The "real-life" validation cohort included 91 consecutive DLBCL NOS cases with available FFPE tissue for T-GEP and IHC, treated with R-CHOP/CHOP-like regimens at the Seragnoli Institute of Hematology and Medical Oncology in Bologna. Success rates of T-GEP was 91% (8 failures); 10 cases were excluded for issues related to histologic classification (non-NOS histology) and 7 additional cases for lack of tissue availability for additional IHC studies. 69 NOS-DLBCL FFPE patient samples from the DLC04 trial were finally included in this study. Patients in the DLCL04 trial were treated according to the

respective treatment protocols, with 30 patients undergoing autologous stem cell transplant (ASCT) consolidation. None of the patients in the real-life cohort underwent ASCT consolidation, according to current treatment guidelines. The OS curve of the real life cohort is depicted in Figure S1. Study algorithm is detailed in figure 1.

Immunohistochemistry

Immunohistochemistry (IHC) was centralized in Bologna for the DLCL04 trial and in Milan for the real-life control group. The IHC results were independently evaluated by 4 expert haematopathologists (CA, SP, SF, VT). Protein expression was recorded as the percentage of positive tumor cells in 10% increments. The antibodies source and dilutions are shown in Table S1.

Tissue microarrays (TMAs) were prepared from paraffin blocks using a precision instrument as previously described [3]. One-mm diameter cores were obtained from representative tumor areas and transferred into a recipient paraffin block, cut to 2-mm thick sections, and transferred on glass slides. At both sites, antigen retrieval was carried on PT-links at 92°C for 5 minutes in EnVision Flex Target Retrieval Solution High pH (Dako Agilent, Glostrup, Denmark). All IHC tests were performed on AutoStainer Plus platforms, using the LSAB+REAL Detection System (Dako Agilent). The IHC preparations were counterstained with Gill’s haematoxylin and mounted in Kaiser’s glycerin. The IHC results were independently evaluated by 4 expert haematopathologists (CA, SAP, SF, VT). In case of discrepant results among the observers, the IHC preparations were jointly reviewed at a multi-head microscope until consensus was reached. Microphotographs were taken using an Olympus BX53 light microscope with an Olympus DP71 camera. Original magnifications are specified in figure legends.

Cell lines

The human DLBCL-derived cell lines SUDHL-4, SUDHL-6, OCI-LY-19, BJAB, U-2932 were obtained from the DSMZ-German Collection of Microorganisms and Cell Cultures, Department of Human and Animal Cell Cultures (Braunschweig, Germany); SUDHL-2, SUDHL-10, OCI-LY-1, OCI-LY-18, DOHH2, SUDHL-5, PFEIFFER, DAUDI were obtained from ATCC (American Type Culture Collection). The DLBCL derived cell lines (HBL-1, TMD8) were provided by Dr. A. Younes (Memorial Sloan Kettering Cancer Center, NY). Cell lines were cultured in RPMI 1640 medium or in Iscove Modified Dulbecco medium supplemented with 10 to 20% heat-inactivated fetal bovine serum, 1% L-glutamine, and penicillin-streptomycin in a humid environment of 5% CO_2 at 37°C.

Genomic data cell line annotations are in line with Cancer Cell Line Encyclopedia (CCLE: https://portals.broadinstitute.org/ccle/about), CBio Portal for Cancer Genomics (https://www.cbioportal.org/study/summary?id=lymphoma_cellline_msk_2020) [4], Derenzini et al. Oncotarget 2015 [5], Hicks et al. Haematologica 2018 [6], Juskevicius et al. Leukemia & Lymphoma 2018 [7], Li et al. J Hematol Oncol 2019 [8].

Inducible Tet-On BCL-2 overexpression system

To overexpress the coding sequence (CDS) of *BCL2* in the SUDHL5 BCL2 negative cell line we used the Cellecta InDOXible Tet-Activated cDNA Lentiviral Expression System (custom Cellecta).

Two lentiviral Vectors, the inducible cDNA, that is under the control of the responsive promoter (TRE), and the transactivator (rtTA), were transduced separately into the SUDHL5 cells, allowing gene overexpression after the tetracycline analog doxocycline induction. The

transactivator vector contains a puromycin resistance gene (Puro) while the TRE-Empty and TRE-BCL2 vectors contain a green fluorescent protein (GFP) mark (Ubi-GFP).

Western blotting

Preparation of cellular protein lysates was performed by using the Cell Signalling lysis buffer (#9803) according to manufacturer`s extraction protocol. A total of 30 ug of protein was denatured in Laemli buffer at 95C for 5 minutes and western immunoblotting was performed using the Biorad system (TGX 4-15% gels). Transfer was performed using the Trans Blot turbo system (Biorad) onto PVDF membranes. Images were acquired by using the BioRad Imaging Chemidoc MP system. Secondary anti-rabbit and anti-mouse HRP-conjugated antibodies were purchased from Biorad (#170-6515, #170-6516).

The following antibodies for western blotting were purchased from cell signalling technology: pCHK1 S345 (#2348), γH2AX S139 (#2577), Cleaved Caspase 3 (#9664), BCL-2 (#4223).

The following antibodies were purchased from Abcam: c-MYC (#32072), lamin B1 (#16048). Beta-Actin (#A5316) and Vinculin (#V9131) were from SIGMA.

Caspase 3/7 activity assay

Cells were seeded in 96-well plates at 25,000 cell/100 µl/well with either vehicle (DMSO 0.1%) or of drugs for 1, 3, 6, 12 and 24 hours. Measurements of caspase activities in cells were performed using the commercially available Caspase-Glo 3/7 Assay (Promega), according to the manufacturer’s instructions.

qPCR assays

Total RNA was extracted with the ZYMO RESEARCH Quick-RNA MiniPrep kit protocol. A total of 1 μg of RNA was converted to cDNA using LunaScript RT SuperMix kit (Bio-Labs). Real-time polymerase chain reaction (PCR) was performed using ABI7500 PCR machine (Applied Biosystem). Primers used are *ACTIN, MYC, BCL-2*.

Primer	Sequence
MYC Fw	TTCGGGTAGTGGAAAACCAG
MYC Rv	CAGCAGCTCGAATTTCTTCC
ACTIN Fw	GAACGGTGGTGTGTCGTTC
ACTIN Rv	GCGTCTCGTCTCGTCTCACT
BCL-2 Fw	GAGTTCGGTGGGGTCATGT
BCL-2 Rv	GCCGGTTCAGGTACTCAGTC

Cell cycle analysis

Single cell suspensions were prepared and fixed with 70% fresh Ethanol for 2 hrs. After fixation, cells were washed twice with 1x PBS, 1% BSA. Cells were then stained with Propidium Iodide (PI Sigma) 10 μg/ml + RNase overnight, protected from light before analysis. Data Acquisition: Flow cytometric data was acquired on a BD FACS Celesta using BD FacsDIVA Software. Analysis was performed using Flow.Jo 9.3.

FISH analysis

FISH studies were conducted on paraffin sections using the following probes: Vysis LSI *MYC* dual color break-apart, Vysis LSI *BCL-2* dual color break-apart, Vysis LSI *BCL-6* dual color break-apart and Vysis LSI IGH/MYC/CEP8 Tri-color FISH probe kit. In brief, the slides were deparaffinized, co-denatured with probe, hybridized overnight, washed and then mounted with DAPI/Antifade. For each probe, 200 interphase nuclei were analyzed to

detect rearrangement and numerical abnormalities. Cut-off values were established for each probe by assessing 10 normal controls (reactive lymph nodes) and choosing values 3SD above the mean. Gains were considered when a pattern of three or four copies of the gene were identified, whereas more than four copies were considered as amplifications.

Additional NanoString methods

Total RNA was extracted from three sections of 15-μm of each FFPE sample using RecoverAll Total Nucleic Acid Isolation Kit for FFPE (Thermo Fisher). Yield and quality of RNA extracted was assessed. Quantitative RNA analysis was performed using NanoDrop ND-1000 Spectrophotometer (NanoDrop Technologies, Rockland, DE, USA). RNA quality was scored according to DV200 values (percentage of RNA fragments ≥200 nucleotides), utilizing the Agilent 2100 BioAnalyzer.

Gene expression was measured on the NanoString nCounter Analysis System (NanoString Technologies, Seattle, WA, USA).Gene expression was measured on the NanoString nCounter Analysis System (NanoString Technologies, Seattle, WA, USA).

Gene expression data were analyzed by the NanoString Company using a modified RUO version of the NanoString Lymphoma Subtyping Test (LST) algorithm to determine the Cell-of-Origin molecular subtype of each sample [9]. The system computes the relative abundance of each mRNA transcript of interest, through a multiplexed hybridization assay and digital readouts of fluorescent barcoded probes, which are hybridized to each transcript. An nCounter CodeSet (NanoString Technologies) containing capture and reporter probes (the latter attached to a color barcode) was hybridized to 200 ng of total RNA for 20 hours at 65 °C, according to the manufacturer's instructions. Hybridized samples were loaded into the nCounter Prep Station for post-hybridization processing. Target mRNA was assessed with nCounter Digital Analyzer.

For T-GEP analyses, the quality control and normalization of NanoString nCounter data were performed using R package NanoStringNorm. The Raw NanoString counts for each gene were subjected to a technical normalization considering positive and negative probes. A normalization factor was calculated by obtaining the geometric mean of the positive controls used for each sample and applied to the raw counts of the nCounter output data to eliminate variability that was unrelated to the samples. The resulting data were normalized again with the geometric mean of the housekeeping genes (*ISY1*, *R3HDM1*, *TRIM56*, *UBXN4* and *WDR55*). Normalized data were log2-transformed for further analyses.

List of genes and target sequences of the 22- gene panel

GeneName ProbeID Comments TargetSeq

1 *UBXN4* NM_014607.3 HOUSEKEEPING
CATCGCGACGGCCAAAAGGAGCGGCGCGGTCTTCGTGGTGTTCGTGGCAGGTGATGATGAACAGTCTACACAGATGGCTGCAAGTTGGGAAGATGATAAA

2 *ISY1* NM_020701.2 HOUSEKEEPING
GGCAAAACATCAGTGTCTGTGGGTAGTTGGAATCTTCAGTTCCTGTGAGCGTCGGCGTCTTCTGGGCCTGTGGAGTTTCTTGGACAGGGGCCGCGGGGCT

3 *R3HDM1* NM_015361.2 HOUSEKEEPING
CCTGTGTTCCCAAGAGAATTACATTATTGACAAAAGACTCCAAGACGAGGATGCCAGTAGTACCCAGCAGAGGCGCCAGATATTTAGAGTTAATAAAGAT

4 *WDR55* NM_017706.4 HOUSEKEEPING
CTACCTCTTCAATTGGAATGGCTTTGGGGCCACAAGTGACCGCTTTGCCCTGAGAGCTGAATCTATCGACTGCATGGTTCCAGTCACCGAGAGTCTGCTG

5 *TRIM56* NM_030961.1 HOUSEKEEPING
GTGGAGGCCGAGGACATTTTCCTGAAGGGCAGGGGTTGGCAACTTTTCAACATGGAGTGCCAAACTGCTAACCCGTCTTCTAGTGTGTGAGAATAGGGAC

6 *MYC* NM_002467.3 ENDOGENOUS
TCGGACACCGAGGAGAATGTCAAGAGGCGAACACACAACGTCTTGGAGCGCCAGAGGAGGAACGAGCTAAAACGGAGCTTTTTTGCCCTGCGTGACCAGA

7 *PIM2* NM_006875.2 ENDOGENOUS
GCCATCCAGCACTGCCATTCCCGTGGAGTTGTCCATCGTGACATCAAGGATGA
GAACATCCTGATAGACCTACGCCGTGGCTGTGCCAAACTCATTGATT

8 *IRF4* NM_002460.1 ENDOGENOUS
GGGCACTGTTTAAAGGAAAGTTCCGAGAAGGCATCGACAAGCCGGACCCTCC
CACCTGGAAGACGCGCCTGCGGTGCGCTTTGAACAAGAGCAATGACTT

9 *TNFRSF13B* NM_012452.2 ENDOGENOUS
TGCAAAACCATTTGCAACCATCAGAGCCAGCGCACCTGTGCAGCCTTCTGCAG
GTCACTCAGCTGCCGCAAGGAGCAAGGCAAGTTCTATGACCATCTCC

10 *S1PR2* NM_004230.2 ENDOGENOUS
TCCCGCCAGGTGGCCTCGGCCTTCATCGTCATCCTCTGTTGCGCCATTGTGGT
GGAAAACCTTCTGGTGCTCATTGCGGTGGCCCGAAACAGCAAGTTCC

11 *MME* NM_000902.2 ENDOGENOUS
GGATTGTAGGTGCAAGCTGTCCAGAGAAAAGAGTCCTTGTTCCAGCCCTATTC
TGCCACTCCTGACAGGGTGACCTTGGGTATTTGCAATATTCCTTTGG

12 *ASB13* NM_024701.3 ENDOGENOUS
GGACACGTAGGCGGTACCACTAAGGTTTTGGTAATGAGCCATTCAAACCGACA
GCAGTGTGAAGGTGTGTCAAGGTGTATATTCTCGTGGCTCGGCATTC

13 *BCL2* NM_000657.2 ENDOGENOUS
GTGAAGCAGAAGTCTGGGAATCGATCTGGAAATCCTCCTAATTTTTACTCCCTC
TCCCCGCGACTCCTGATTCATTGGGAAGTTTCAAATCAGCTATAAC

14 *CYB5R* NM_016229.3 ENDOGENOUS
CCATGTCTTAGGGCTTCCTGTAGGTAACTATGTCCAGCTCTTGGCAAAAATCGA
TAATGAATTGGTGGTCAGGGCTTACACCCCTGTCTCCAGTGATGAT

15 *MAML3* NM_018717.4 ENDOGENOUS
TGGAAGCCATCAACAATTTGCCCAGTAACATGCCACTGCCTTCAGCTTCTCCTC
TTCACCAACTTGACCTGAAACCTTCTTTGCCCTTGCAGAACAGTGG

16 *SERPINA9* NM_001042518.1 ENDOGENOUS
CCACTAAATCCTAGGTGGGAAATGGCCTGTTAACTGATGGCACATTGCTAATG
CACAAGAAATAACAAACCACATCCCTCTTTCTGTTCTGAGGGTGCAT

17 *MYBL1* XM_034274.14 ENDOGENOUS
CTCCTTTTAAGAATGCGCTTGCTGCTCAGGAGAAAAAATATGGACCTCTTAAAA
TTGTGTCCCAGCCACTTGCTTTCTTGGAAGAAGATATTCGGGAAGT

18 *RAB7L1* NM_001135664.1 ENDOGENOUS
CATTTGAATTGTCTCCTGACTACTGTCCAGTAAGGAGGCCCATTGTCACTTAGA
AAAGACACCTGGAACCCATGTGCATTTCTGCATCTCCTGGATTAGC

19 *LIMD1* NM_014240.2 ENDOGENOUS
AAGGCAAGTCTCAGGAACCCATGCAGGTACATCGCTTGCACCTGTTTTTAGCT
TATTTAATGACGGGCTTTTGGGAAGAGCTGCCCGCATACTGAGAGAC

20 *ITPKB* NM_002221.3 ENDOGENOUS GGTTTGCGCCTCTGGGCATGTAGTCTACACAGGACCTGAGAATCTGAGAAACTGCAGCCGCACGGTTGTTTATGGAGCTTTGGGCGGGGGCTGAGCCCGC

21 *CREB3L2* NM_001253775.1 ENDOGENOUS CGCACTTCTCAGAACTTCTGGATGAGTTTTCCCAGAACGTCTTGGGTCAGCTCCTGAATGATCCTTTCCTCTCAGAGAAGAGTGTGTCAATGGAGGTGGA

22 *CCDC50* NM_174908.3 ENDOGENOUS AGGACATAGCTCGCCTTTTGCAAGAAAAGGAGTTACAGGAAGAGAAAAAGAGAAAGAAACACTTTCCAGAGTTCCCTGCAACCCGTGCTTATGCAGATAG

Additional HTS methods and statistical analysis

For the synergy assessment studies, a compound matrix was prepared in which compound 1 was titrated against compound 2 at 2.5 µM in a doubling dilution series and transferred into a 1536-well microtiter assay plate. Internal controls for each assay plate were dispensed as previously described [10]. To start the assay, cells were seeded at 1,000 cells per well in 8 µL of complete RPMI medium and incubated for 2 days followed by 1 µL Alamar Blue for an additional day at 37°C. Plates were imaged on the LEADseeker™ Multimodality Imaging System (GE Healthcare, Piscataway, NJ) and resulting files were deconvoluted to obtain fluorescence values associated with each drug.

The drugs were tested for activity both as single agents and in combinations on multiple cell lines through the MSKCC high throughput screening core facility (HTSCF). The residual cell viability post treatment with specific drug combinations was assessed in an Alamar Blue (AB) assay using the LEADseeker Multimodality Imaging System (GE Healthcare, Piscataway, NJ). The data were converted into percent inhibitions conferred by each combination relative to both the high (1% DMSO v/v) and the low (1 µM killer mix) control averages (µ). “killer mix” consists of a HTSCF proprietary mixture of cytotoxic compounds. The percent inhibitions were defined as:

$$\%\mathbf{inhib}_i = (\mu_{\text{high control}} - \text{value}_i)/(\mu_{\text{high control}} - \mu_{\text{low control}}) \times 100$$

Since the percent inhibition is derived from data measured with error some of the computed numbers can fall outside the [0, 100]% interval. We replaced such valued by the appropriate boundary value. We used the average percent inhibition of the replicates at a dose level (single agent or combination) as the activity at that dose level.

In order to evaluate whether a drug combination shows synergy we compared the observed activity at the combination at that level to the expected activity under Bliss independence model. The expected activity can be written as

$$P(Inh|A,B) = 1 - P\left(\overline{Inh}\middle|A,B\right) = 1 - P\left(\overline{Inh}\middle|A\right) \times P\left(\overline{Inh}\middle|B\right)$$
$$= 1 - [1 - P(Inh|A)] \times [1 - P(Inh|B)]$$

where A and B are the two drugs, Inh and $\overline{Inh}$ denote inhibited and not inhibited respectively [11,12]. The observed can be compared to the expected activity using a simple difference where values around 0 represent additive relationship, large positive values represent synergy and large negative values antagonism. However the same magnitude of the difference represents different relative change depending on the expected activity. Thus we also use a log-odds measure given as $\log\{[P_o(1-P_e)]/[P_e(1-P_o)]\}$ where P_o and P_e are observed and expected activities. For each drug combination we generated a heatmap for the observed inhibition, the difference between observed and expected inhibition and the log-odds of the observed to expected inhibition. The observed and expected inhibition values were rescaled prior to calculating the log-odds by the function (0.8 * observed/expected + 0.1), this was done in order to adjust for instances in which the observed or expected inhibition was 0 or 1. These numbers are binned into intervals suitable for each scale and color coded for simple visualization of the combined activity. For comparing drug combinations we combined the log-odds data across all cell lines and used the medians to rank the combinations. The data are shown as boxplots. All analyses were done using R programming language [13].

8-OHdG ELISA assay

Determination of 8-hydroxy-2 deoxyguanosine (8-OHdG) levels on purified cellular DNA was performed by using the competitive enzyme-linked immunosorbent assay (ELISA) kit (ab201734, Abcam) following the manufacturer's protocol.

Cells purified DNA was digested using nuclease P1, treated with alkaline phosphatase and added to an 8-OHdG-coated 96-well plate that was detected with horseradish peroxidase conjugated 8-OHdG antibody. A colored signal was obtained after incubation with tetramethylbenzidine substrate, and the absorbance was measured at 450 nm, using a multiplate reader (GloMax® Discover Microplate Reader). The concentrations were calculated according to standard curve obtained from the OD values and final values were then normalized to the control sample concentration (ng/ml).

In vitro proliferation assay and combination index calculation.

Cells were seeded in 96-well plates at 25,000 cell/100 µl/well with either DMSO 0.1% or increasing concentrations of drugs for the indicated time. Cell viability was assessed by adding Cell Titer Glo reagent (Promega) to the culture medium, according to manufacturer`s instructions. Replicates were averaged for the combination index analysis. The selected combinations were tested for nature of effect using the Chou-Talalay (CT) method [14], which enables quantification of drug outcomes as a combination index (CI); CI=1 shows additivity, CI<1 shows synergism, and CI>1 shows antagonism.

In vivo studies

Cells (10^6) were xenografted via tail vein injection into 6- to 8-week-old female NSG mice (Charles River, Italy). Tumour growth was monitored three times per week by whole-body imaging on an IVIS Lumina III platform.

Mice were bred and housed under pathogen-free conditions in the animal facilities at the European Institute of Oncology–Italian Foundation for Cancer Research (FIRC) Institute of Molecular Oncology (IEO–IFOM, Milan, Italy) campus.

Briefly, mouse were IP injected with 150 mg/kg of XenoLight D-Luciferin - K+ Salt Bioluminescent Substrate (PerkinElmer). After 10 minutes, animals were anaesthetized with isofluorane apparatus and images acquired using Living Image Software, version 4.2 (Caliper Life Sciences). Radiant efficiency was calculated on the basis of the epifluorescence signal, as indicated in the user manual. Treatment started about 20 days from tumor injection, when a bioluminescence signal was detectable in each individual mouse.

In the first set of experiments, 5 mice per group were randomized into control and treatment arms receiving either a vehicle (control group) or different dose levels of the compounds, specifically: i) Prexasertib 5 and 10 mg/kg, subcutaneous, BID, 3 times a week, and ii) Venetoclax 25, 50 and 75 mg/kg, by oral gavage, for 5 days on- 2 days off for a total of 21 days (venetoclax data not shown). In another set of experiments, combination studies were performed as follows: Vehicle, Prexasertib 2,50 and 1,25 mg/kg, Venetoclax 50 mg/kg, and the combinations (Prexasertib 1.25 mg/kg BID 3 days/week + Venetoclax 50 mg/kg 5 days on - 2 days off; Prexasertib 2.5 mg/kg BID 3 days/week + Venetoclax 50 mg/kg 5 days on- 2 days off). Five mice per group were used. For combination experiments, with a sample size of 5 mice per group, we can achieve 90% power to detect a difference of overall survival at a significance level of 0.05 with log-rank test, assuming a survival rate of 20% at day 80 in the control groups, and 100% in the experimental group.

Prexasertib was prepared in 20% Captisol™, venetoclax was dissolved in 60% Phosal 50 PG, 30% polyethylene glycol, and 10% ethanol. Solutions were freshly prepared from dry powder just before each injection. Tumors growth and body weight were assessed three times weekly. After 3 weeks of treatment and after 6 hours from last dose tumor samples were collected for immunoblotting.

Mice were observed daily throughout the treatment period for signs of morbidity/mortality.

Supplementary tables

Table S1.

Antibody	Source	Clone	Diluition
γH2AX S139	Cell Signaling 2577	-	1:25
8-OhDG	Abcam	N45.1	1:200
BCL-2	Dako	124	1:100
c-MYC	Abcam	Y69	1:100

Table S2.

**DLCL04 (N=69)**	Patients N°	Hazard Ratio (95%CI)	P-value
MYC/BCL-2 DE Nano			
DE	18	1	0.02
Non-DE	51	0.35 (0.11-1.08)	
MYC/BCL-2 DE IHC			
DE	21	1	0.11
Non-DE	48	0.48 (0.17-1.35)	
**Real-Life Cohort (N=66)**	Patients N°	Hazard Ratio (95%CI)	P-value
MYC/BCL-2 DE Nano			
DE	17	1	0.01
Non-DE	49	0.39 (0.14-1.09)	
MYC/BCL-2 DE IHC			
DE	17	1	0.39
Non-DE	49	0.77 (0.28-2.12)	

MYC/BCL-2 Nano (MYC and BCL-2 mRNA levels assessed by NanoString profiling)

DE (double expresser), non-DE (non-double expresser)

Supplementary Figures

Figure S1

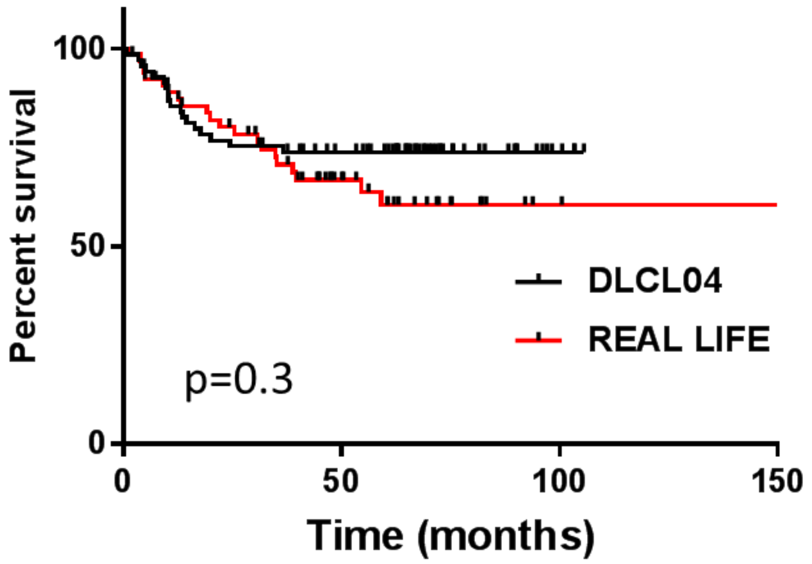

Figure S2

A

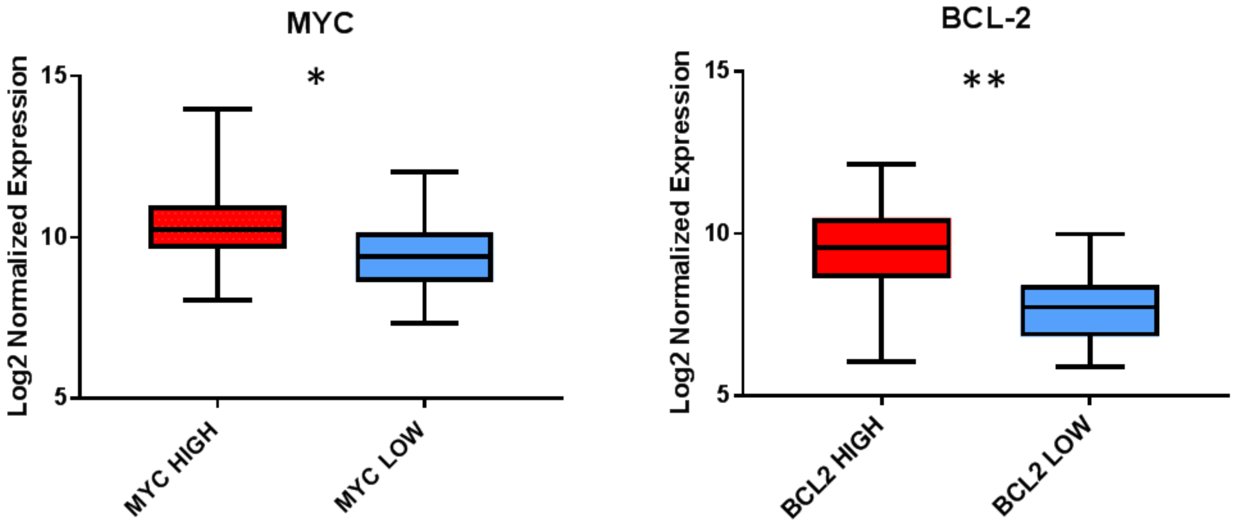

MYC
BCL-2
Log2 Normalized Expression
15
10
5
*
**
MYC HIGH
MYC LOW
BCL2 HIGH
BCL2 LOW

B

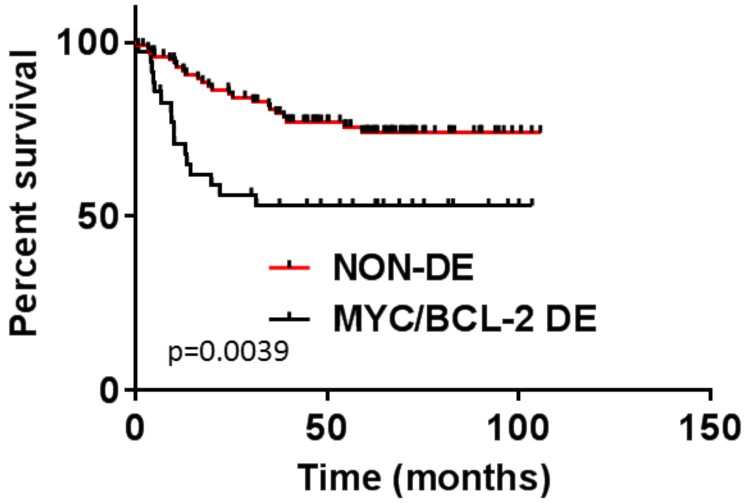

Percent survival
100
50
0
NON-DE
MYC/BCL-2 DE
p=0.0039
0
50
100
150
Time (months)

Figure S3

A

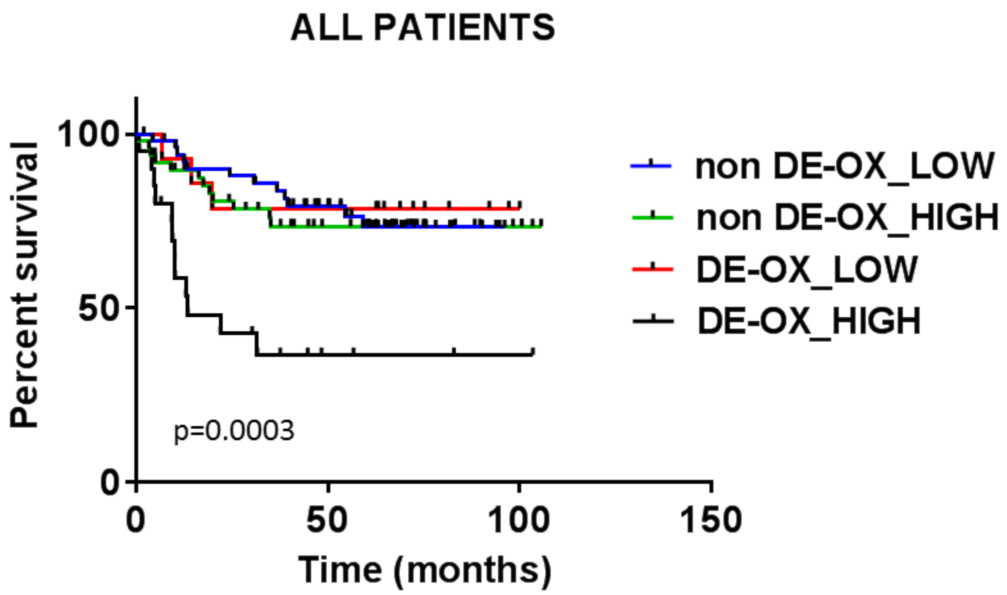
ALL PATIENTS
Percent survival
100
50
0
0
50
100
150
Time (months)
non DE-OX_LOW
non DE-OX_HIGH
DE-OX_LOW
DE-OX_HIGH
p=0.0003

B

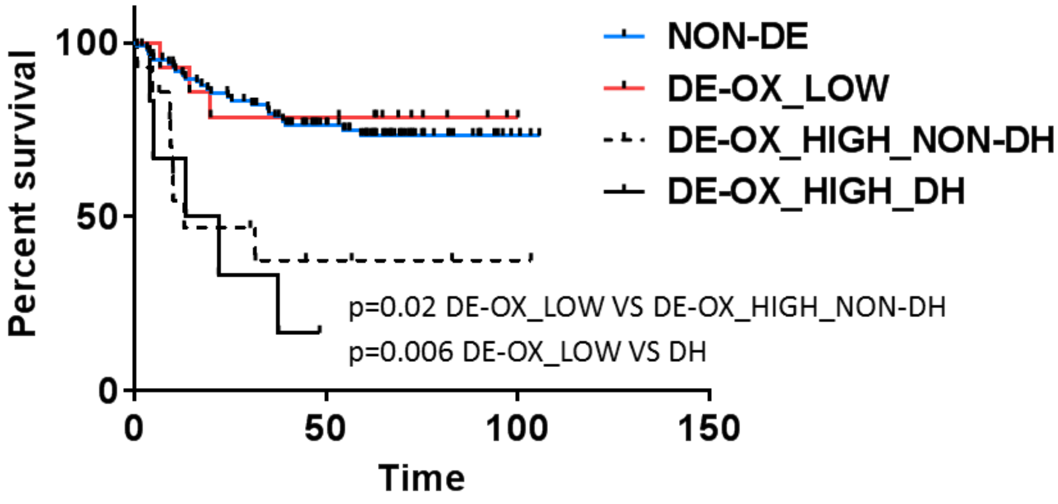
Percent survival
100
50
0
0
50
100
150
Time
NON-DE
DE-OX_LOW
DE-OX_HIGH_NON-DH
DE-OX_HIGH_DH
p=0.02 DE-OX_LOW VS DE-OX_HIGH_NON-DH
p=0.006 DE-OX_LOW VS DH

Figure S4

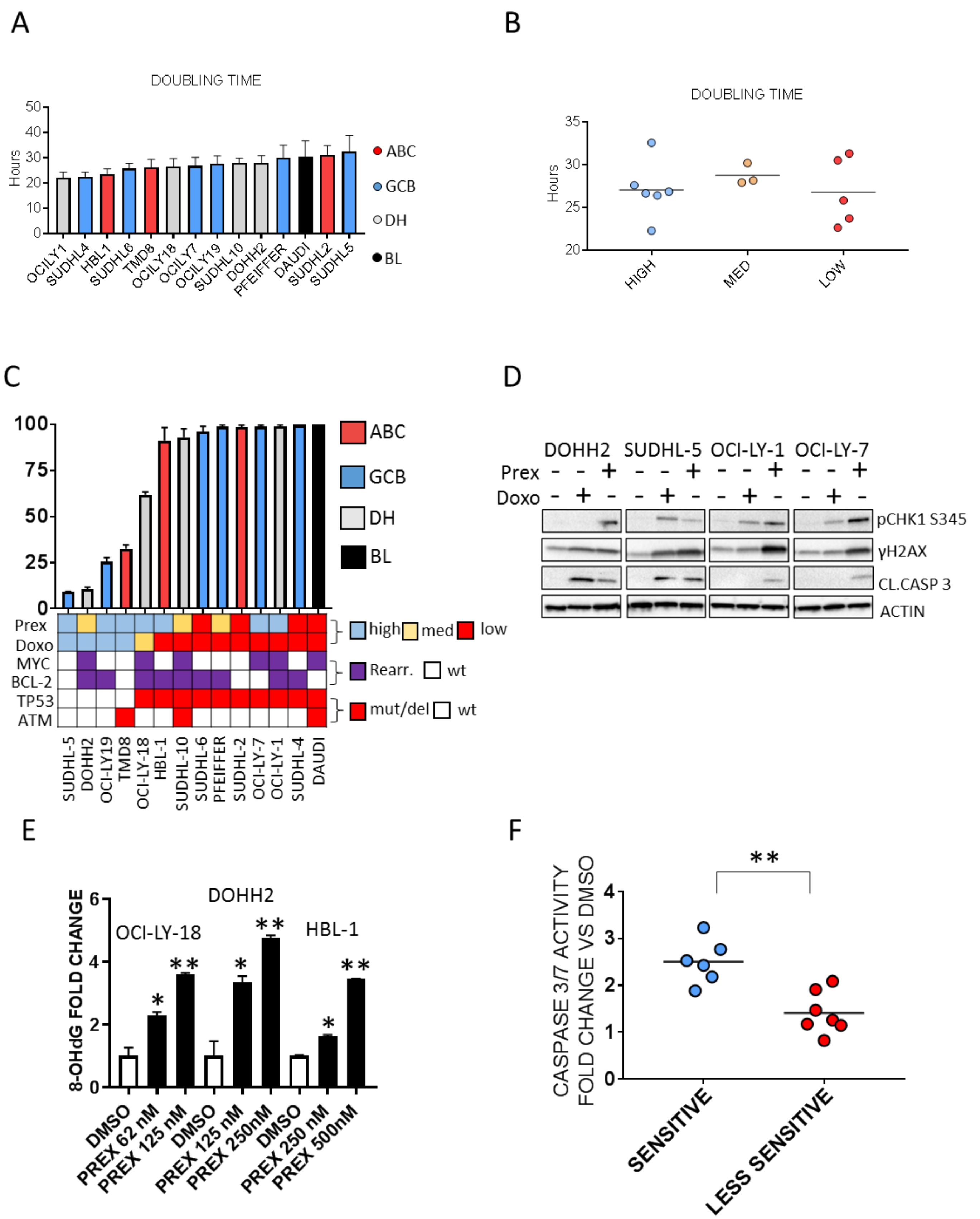
A
DOUBLING TIME
Hours
OCILY1
SUDHL4
HBL1
SUDHL6
TMD8
OCILY18
OCILY7
OCILY19
SUDHL10
DOHH2
PFEIFFER
DAUDI
SUDHL2
SUDHL5
ABC
GCB
DH
BL
B
DOUBLING TIME
Hours
HIGH
MED
LOW
C
ABC
GCB
DH
BL
Prex
Doxo
MYC
BCL-2
TP53
ATM
high
med
low
Rearr.
wt
mut/del
wt
SUDHL-5
DOHH2
OCI-LY19
TMD8
OCI-LY-18
HBL-1
SUDHL-10
SUDHL-6
PFEIFFER
SUDHL-2
OCI-LY-7
OCI-LY-1
SUDHL-4
DAUDI
D
DOHH2
SUDHL-5
OCI-LY-1
OCI-LY-7
Prex
Doxo
pCHK1 S345
γH2AX
CL.CASP 3
ACTIN
E
DOHH2
OCI-LY-18
HBL-1
8-OHdG FOLD CHANGE
DMSO
PREX 62 nM
PREX 125 nM
DMSO
PREX 125 nM
PREX 250nM
DMSO
PREX 250 nM
PREX 500nM
F
CASPASE 3/7 ACTIVITY
FOLD CHANGE VS DMSO
SENSITIVE
LESS SENSITIVE

Figure S5

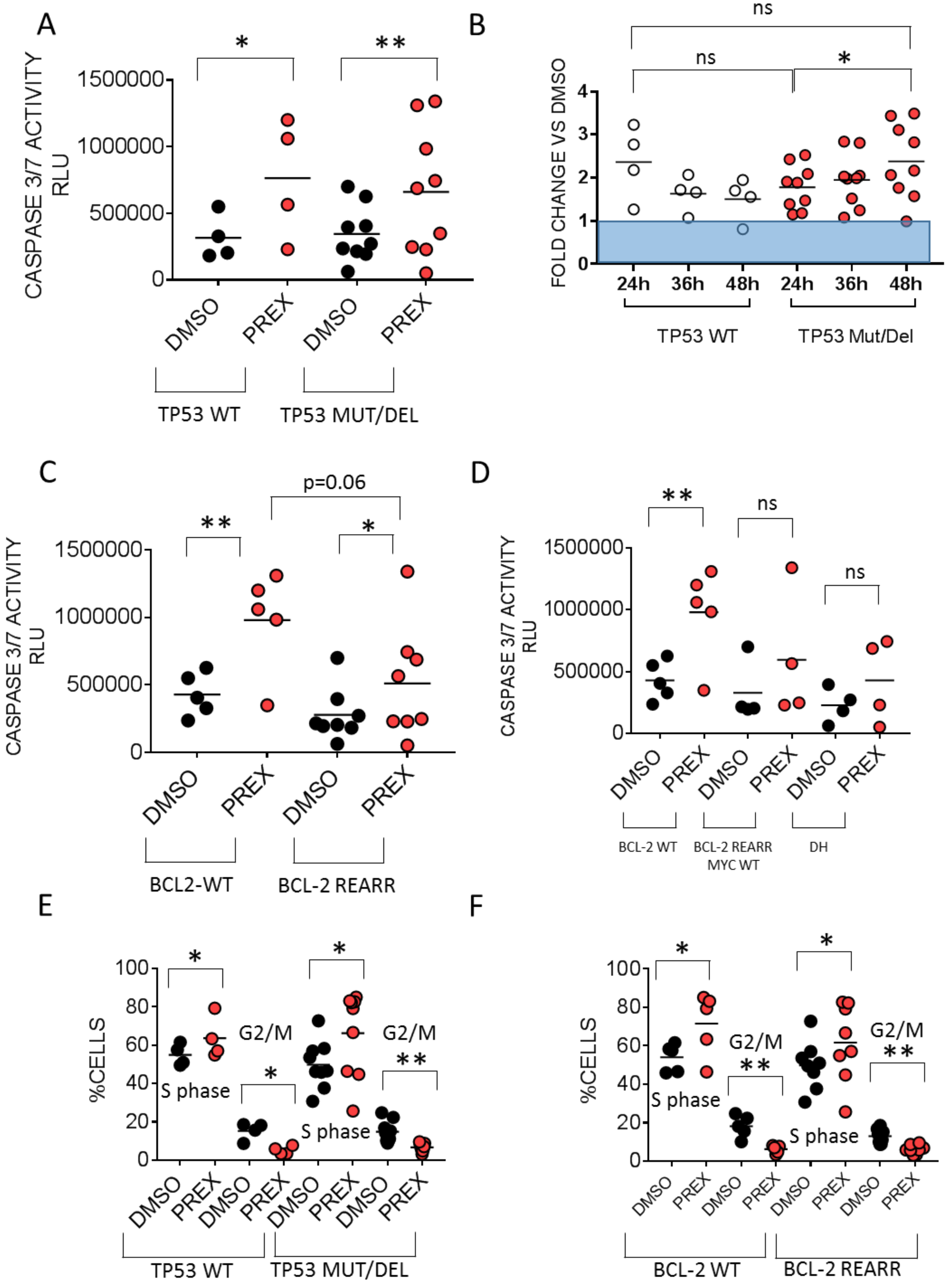

A
CASPASE 3/7 ACTIVITY RLU
1500000
1000000
500000
0
*
**
DMSO
PREX
DMSO
PREX
TP53 WT
TP53 MUT/DEL
B
FOLD CHANGE VS DMSO
ns
ns
*
4
3
2
1
0
24h
36h
48h
24h
36h
48h
TP53 WT
TP53 Mut/Del
C
CASPASE 3/7 ACTIVITY RLU
p=0.06
**
*
1500000
1000000
500000
0
DMSO
PREX
DMSO
PREX
BCL2-WT
BCL-2 REARR
D
CASPASE 3/7 ACTIVITY RLU
**
ns
ns
1500000
1000000
500000
0
DMSO
PREX
DMSO
PREX
DMSO
PREX
BCL-2 WT
BCL-2 REARR MYC WT
DH
E
%CELLS
100
80
60
40
20
0
*
G2/M
*
S phase
*
*
G2/M
**
S phase
DMSO
PREX
DMSO
PREX
DMSO
PREX
DMSO
PREX
TP53 WT
TP53 MUT/DEL
F
%CELLS
100
80
60
40
20
0
*
G2/M
**
S phase
*
G2/M
**
S phase
DMSO
PREX
DMSO
PREX
DMSO
PREX
DMSO
PREX
BCL-2 WT
BCL-2 REARR

Figure S6

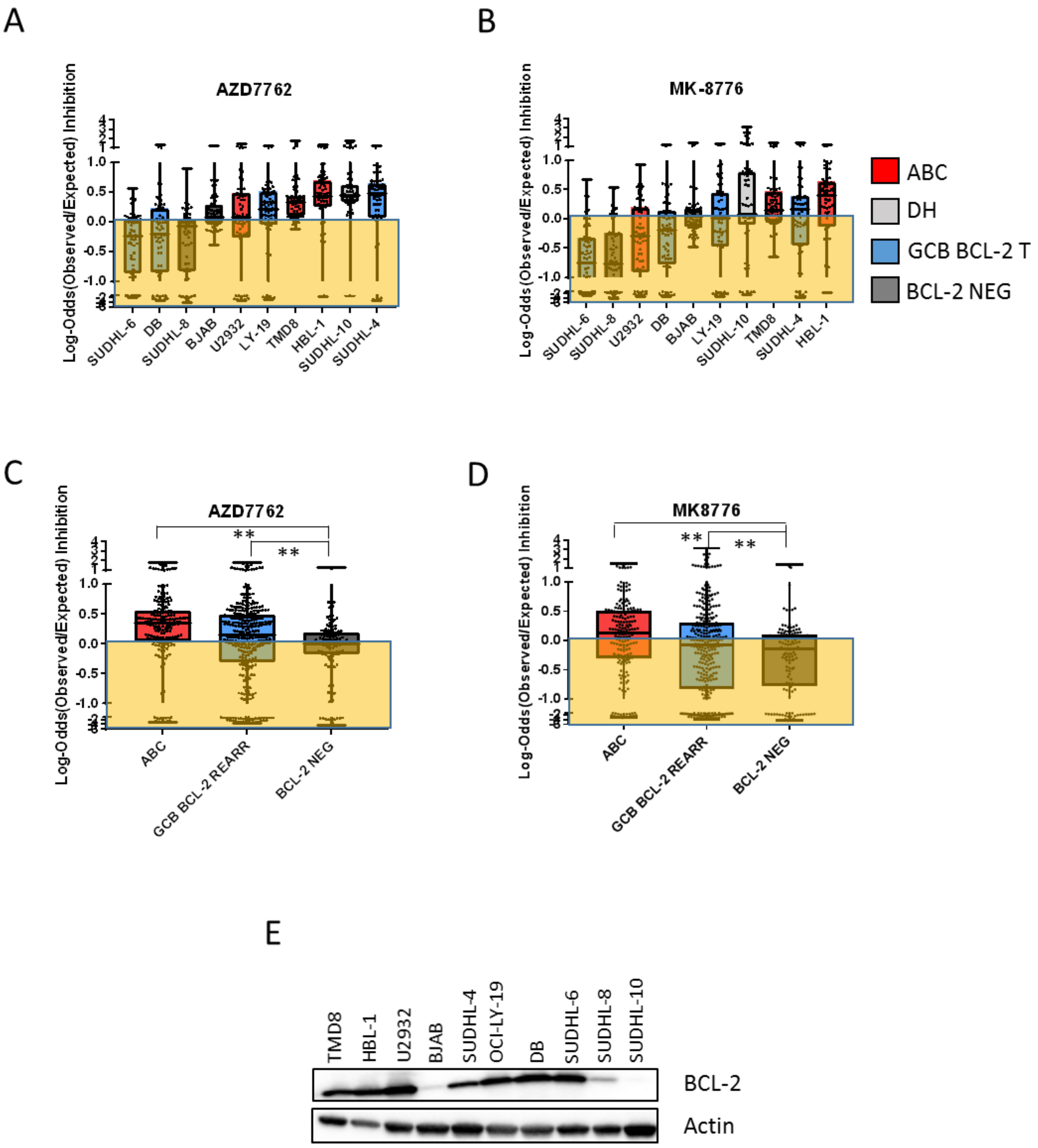
A
B
AZD7762
MK-8776
Log-Odds(Observed/Expected) Inhibition
1.0
0.5
0.0
-0.5
-1.0
SUDHL-6
DB
SUDHL-8
BJAB
U2932
LY-19
TMD8
HBL-1
SUDHL-10
SUDHL-4
ABC
DH
GCB BCL-2 T
BCL-2 NEG
C
D
AZD7762
MK8776
**
**
ABC
GCB BCL-2 REARR
BCL-2 NEG
E
TMD8
HBL-1
U2932
BJAB
SUDHL-4
OCI-LY-19
DB
SUDHL-6
SUDHL-8
SUDHL-10
BCL-2
Actin

Figure S7

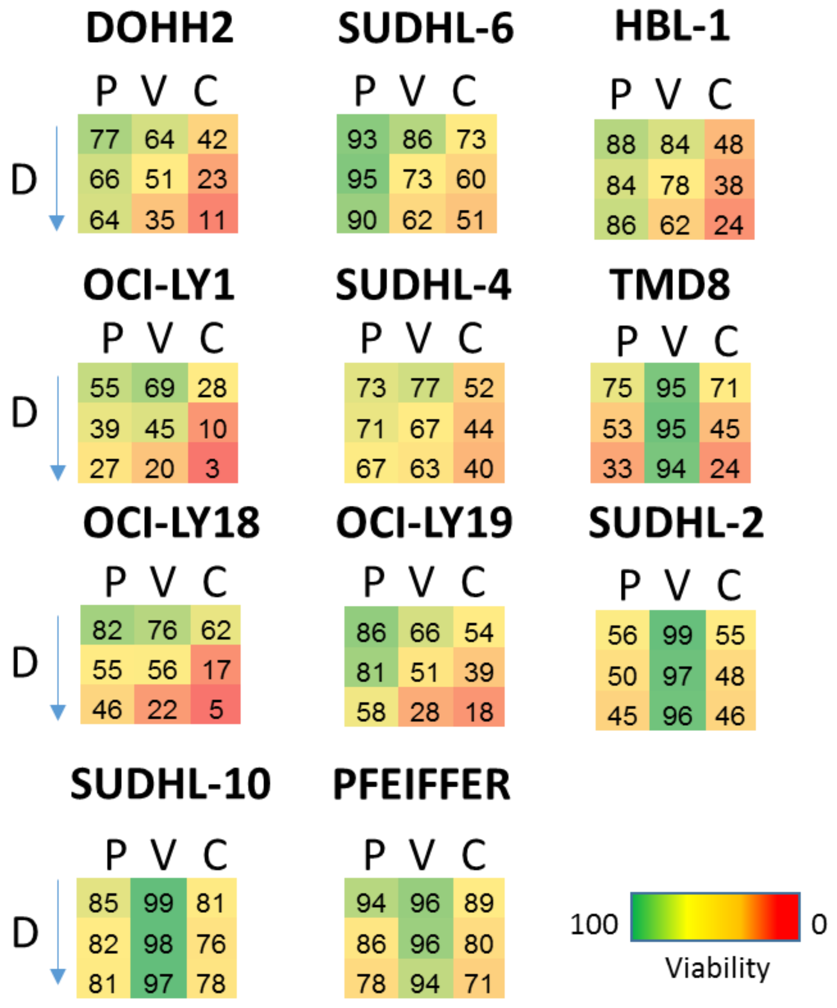
DOHH2
P V C
D
77 64 42
66 51 23
64 35 11
SUDHL-6
P V C
93 86 73
95 73 60
90 62 51
HBL-1
P V C
88 84 48
84 78 38
86 62 24
OCI-LY7
P V C
93 99 96
74 98 76
45 98 50
OCI-LY1
P V C
D
55 69 28
39 45 10
27 20 3
SUDHL-4
P V C
73 77 52
71 67 44
67 63 40
TMD8
P V C
75 95 71
53 95 45
33 94 24
SUDHL-5
P V C
81 99 85
55 98 58
37 97 37
OCI-LY18
P V C
D
82 76 62
55 56 17
46 22 5
OCI-LY19
P V C
86 66 54
81 51 39
58 28 18
SUDHL-2
P V C
56 99 55
50 97 48
45 96 46
SUDHL-10
P V C
D
85 99 81
82 98 76
81 97 78
PFEIFFER
P V C
94 96 89
86 96 80
78 94 71
100
0
Viability

Figure S8

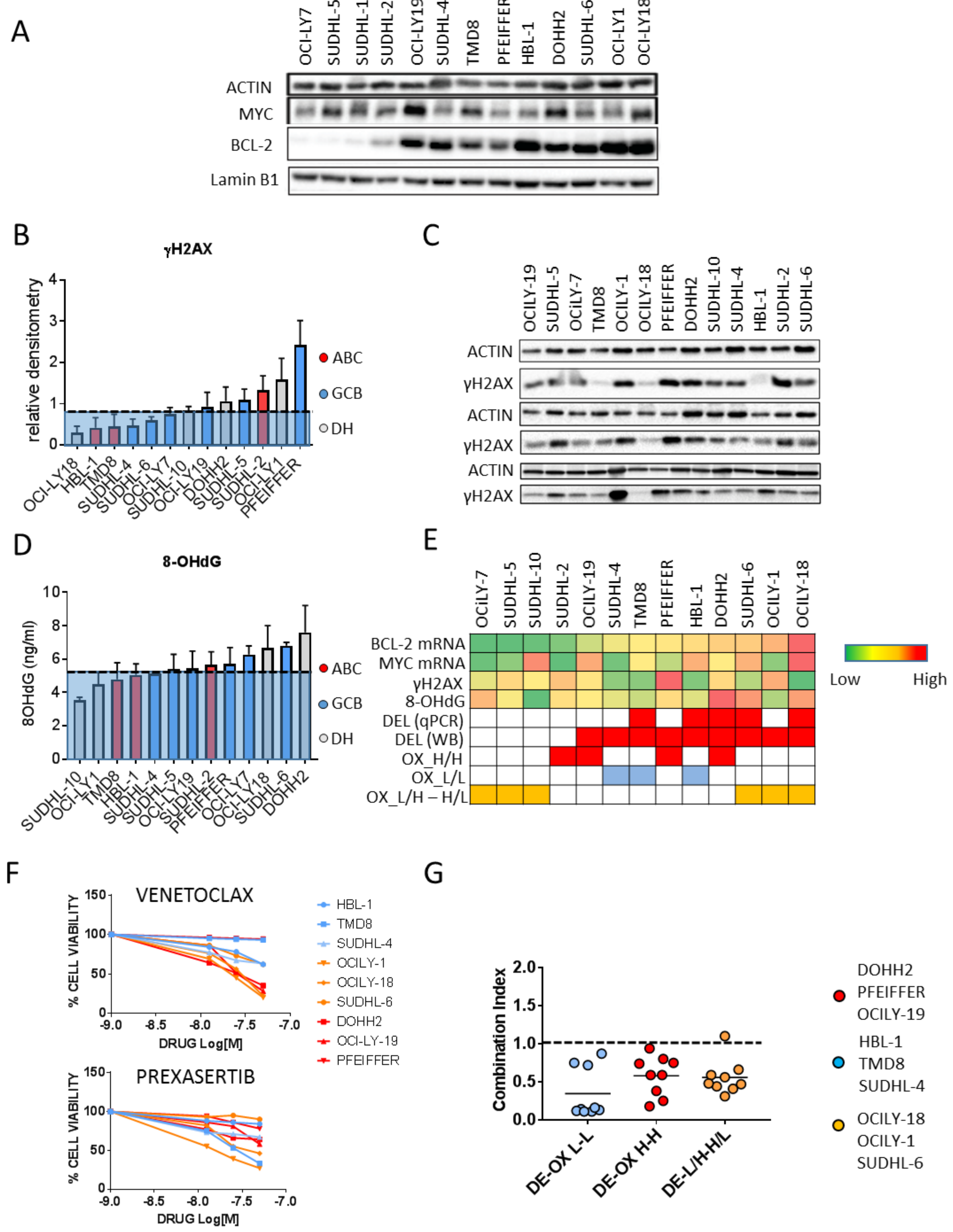
A
OCI-LY7
SUDHL-5
SUDHL-10
SUDHL-2
OCI-LY19
SUDHL-4
TMD8
PFEIFFER
HBL-1
DOHH2
SUDHL-6
OCI-LY1
OCI-LY18
ACTIN
MYC
BCL-2
Lamin B1
B
γH2AX
relative densitometry
ABC
GCB
DH
C
ACTIN
γH2AX
D
8-OHdG
8OHdG (ng/ml)
E
BCL-2 mRNA
MYC mRNA
γH2AX
8-OHdG
DEL (qPCR)
DEL (WB)
OX_H/H
OX_L/L
OX_L/H – H/L
Low
High
F
VENETOCLAX
PREXASERTIB
% CELL VIABILITY
DRUG Log[M]
HBL-1
TMD8
SUDHL-4
OCILY-1
OCILY-18
SUDHL-6
DOHH2
OCI-LY-19
PFEIFFER
G
Combination Index
DE-OX L-L
DE-OX H-H
DE-L/H-H/L
DOHH2
PFEIFFER
OCILY-19
HBL-1
TMD8
SUDHL-4
OCILY-18
OCILY-1
SUDHL-6

Figure S9

A

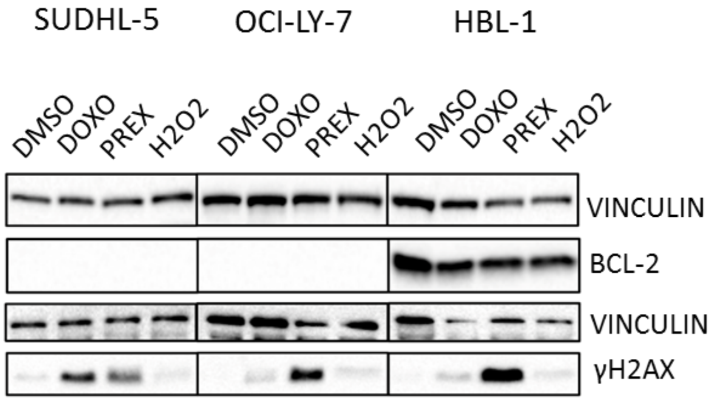
SUDHL-5
OCI-LY-7
HBL-1
DMSO
DOXO
PREX
H2O2
DMSO
DOXO
PREX
H2O2
DMSO
DOXO
PREX
H2O2
VINCULIN
BCL-2
VINCULIN
γH2AX

B

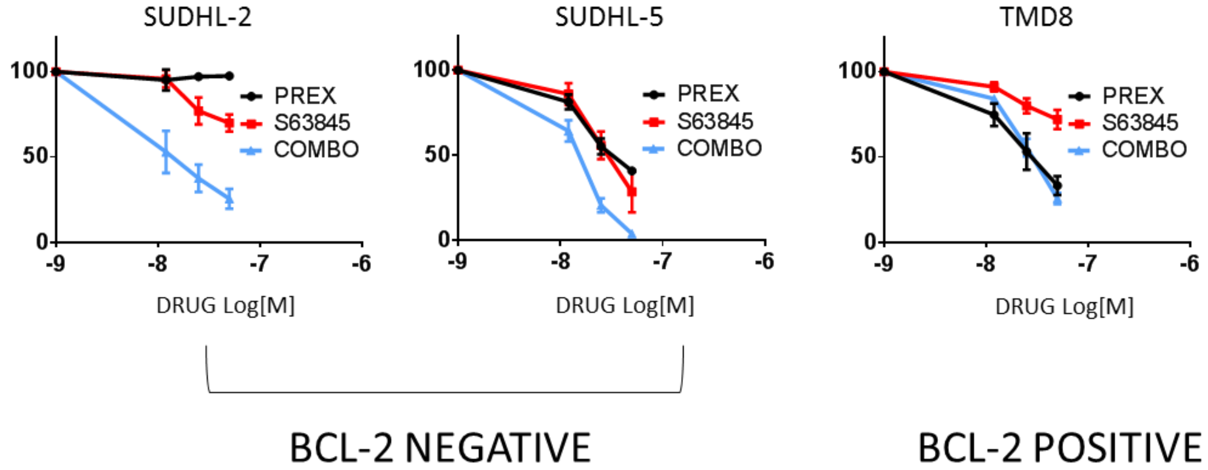
SUDHL-2
SUDHL-5
TMD8
100
50
0
-9
-8
-7
-6
PREX
S63845
COMBO
DRUG Log[M]
BCL-2 NEGATIVE
BCL-2 POSITIVE

Figure S10

A

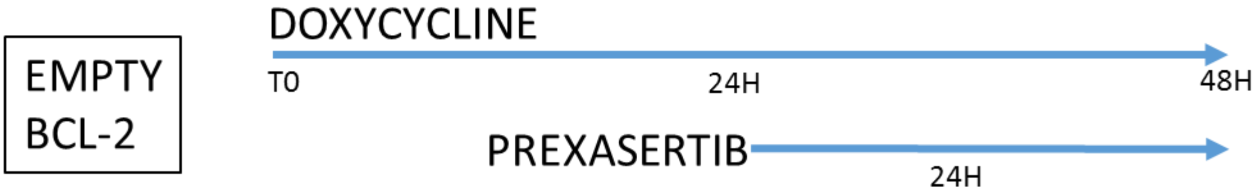

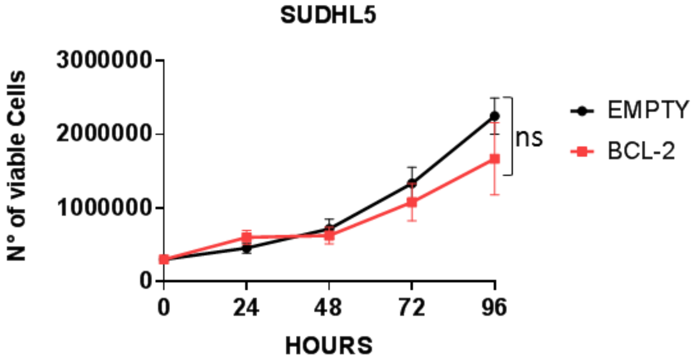

B

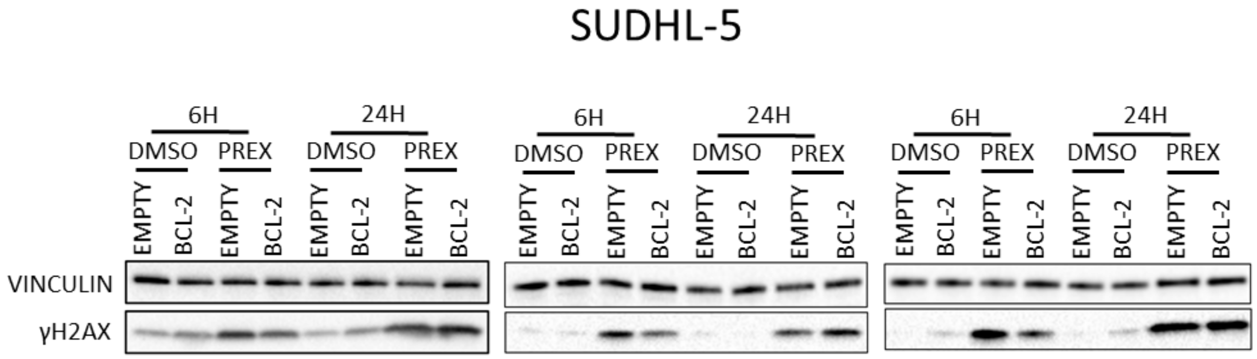

Figure S11

A

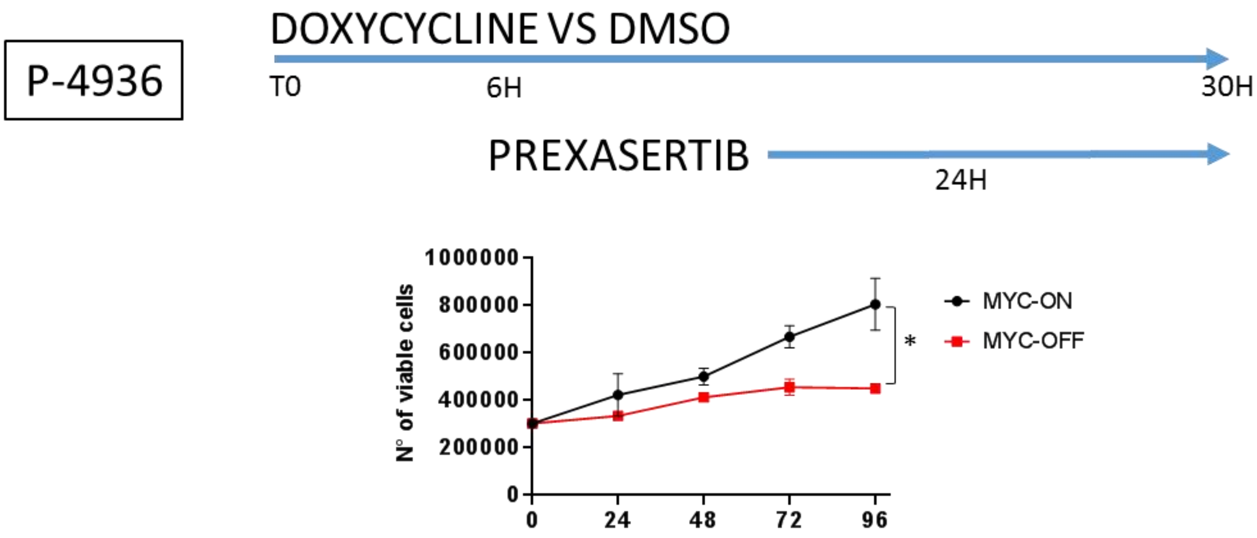

B

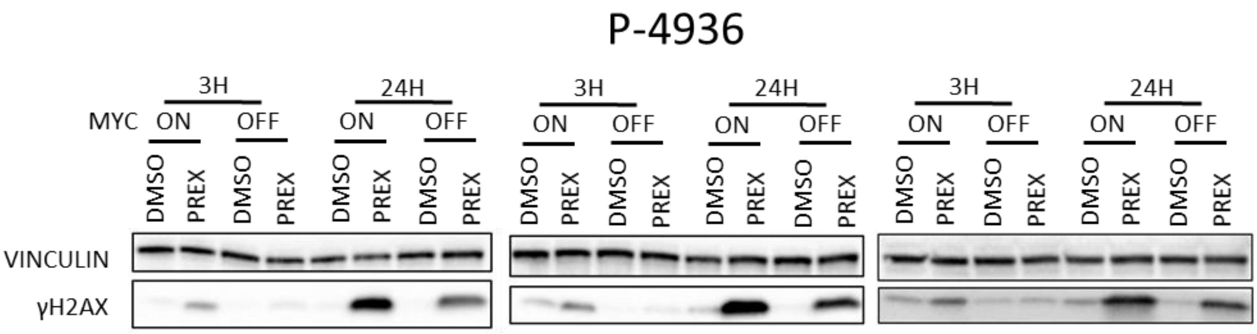

C

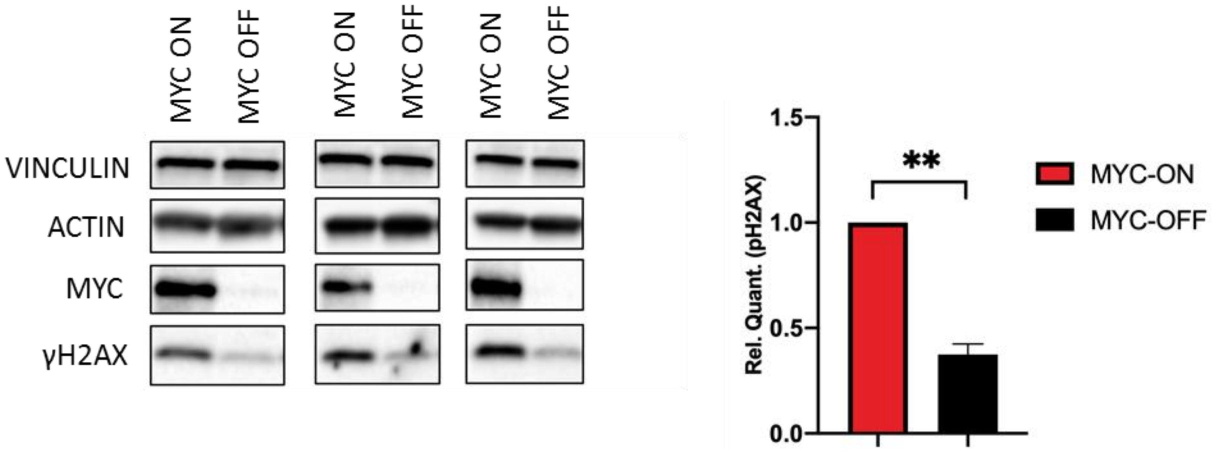

Figure S12

A

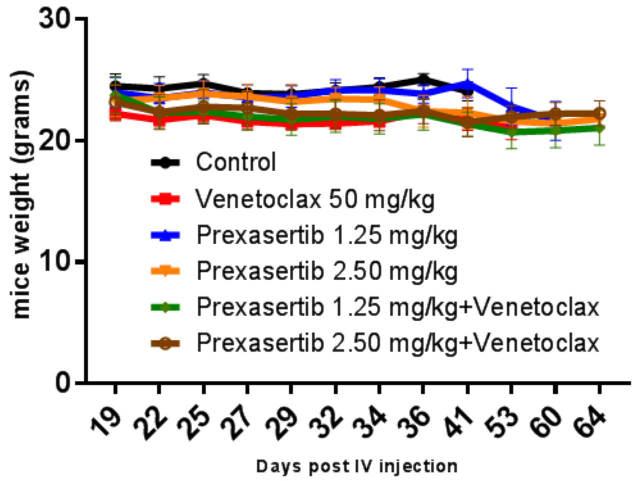

B

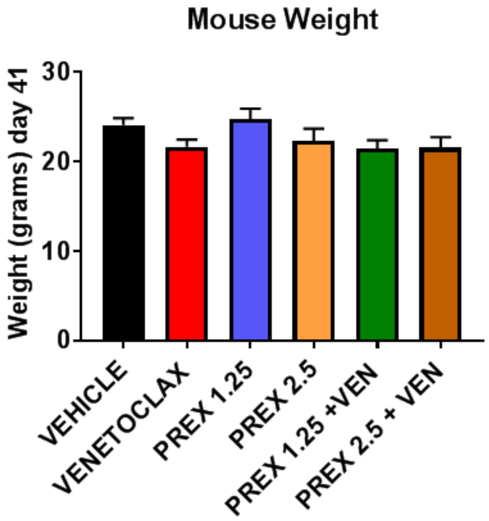

Supplementary Figures Legends

Figure S1. Overall survival of the discovery and validation cohorts.

Overall survival curves of the discovery and validation cohorts, showing similar outcomes in the 2 cohorts. P values were calculated with the log rank test.

Figure S2. MYC and BCL-2 status and outcome

A) Box plot graphs showing significant correlation and concordance between NanoString and immunohistochemistry in the determination of BCL-2 and MYC levels. mRNA levels detected by NanoString in the BCL-2 and MYC negative and positive subgroups as classified by immunohistochemistry (applying a standard 50% and 40% cut-off for BCL-2 and MYC respectively) are represented here. P value were calculated with the Student's T test, * $p < 0.05$, ** $p< 0.01$.
B) Overall survival curve of MYC/BCL-2 double expressers as assessed by T-GEP vs non-double expressers in the whole patients cohort (discovery+validation). P values were calculated with the log rank test.

Figure S3. Integration of MYC/BCL-2 status with γH2AX and 8-OHdG for outcome prediction.

A) Survival analysis integrating MYC/BCL-2 status assessed by T-GEP with γH2AX and 8-OhDG levels assessed by immunohistochemistry in the whole patient cohort (discovery+validation). P values were calculated with the log rank test.

B) OS curves of non-DE (non-double expresser DLBCL) cases vs DE-OX_low vs DE-OX_high_non-DH (DE-OX-high non double hit), vs DE-OX_high_DH (DE-OX_high double hit) are compared. P values were calculated with the log rank test.

Figure S4. Differential activity of Prexasertib and Doxorubicin in DLBCL cell lines and effects of Prexasertib on oxidative DNA damage.

A) Bar graph showing average cell doubling time in our panel of aggressive B-cell lymphoma cell lines. Error bars represent standard error of the mean (SEM) of triplicate measurements.

B) Scatter plot showing cell doubling time according to Prexasertib sensitivity (high vs med vs low) in our panel of B-cell lymphoma cell lines. Each point represents the mean of 3 indepndent experiments.

C) Bar graph showing percentages of cell viability of different B-cell lymphoma cell lines treated with Doxorubicin 125 nM for 24 hours. Below the graph, a heat map showing 1- information on Prexasertib (Prex) and Doxorubicin (Doxo) sensitivity defined as: high > 50% inhibition of cell viability; med 25-50% inhibition; low < 25% inhibition. 2- annotation regarding *MYC, BCL-2, TP53, ATM* status in all cell lines (rearr.: rearrangement; wt: wild type; mut/del: mutation/deletion). See supplemental methods for detailed references of genomic annotations. Error bars represent standard error of the mean (SEM) of triplicate experiments.

D) Representative western blot assays depicting the effects of 24-hour incubation with DMSO, 125 nM Prexasertib or 125 nM Doxorubicin, on γH2AX, pCHK1 S345 and cleaved caspase 3 (CL. CASP 3) expression levels in DLBCL cell lines showing high and intermediate sensitivity to both Prexasertib and Doxorubicin (SUDHL-5

and DOHH2) vs cell lines showing high sensitivity to Prexasertib but low sensitivity to Doxorubicin (OCI-LY-1, OCI-LI-7).

E) Bar graph showing 8-OhDG levels in DNA extracted from OCI-LY-18, DOHH2 and HBL-1 cells treated with DMSO or the indicated doses of Prexasertib (PREX) for 6 hours. Differences between groups were calculated with the Student T test. * $p < 0.05$, ** $p < 0.01$. Error bars represent standard error of the mean (SEM) of triplicate experiments.

F) Scatter plot representing the effects of 125 nM Prexasertib on apoptosis in DLBCL cell lines: highly sensitive (high sensitivity) vs less sensitive (medium or low sensitivity). Cells were incubated with Prexasertib for 24 hours and caspase 3/7 activation assessed with the Caspase Glo 3/7 assay system (Promega™). Each point represents the mean of 3 independent experiments. P value was calculated with the paired Student's T test.

Figure S5. Effects of Prexasertib on apoptosis and cell cycle in DLBCL cell lines according to *BCL-2* and *TP53* status.

A) Scatter plot representing the effects of 125 nM Prexasertib on apoptosis in DLBCL cell lines according to *TP53* status (WT vs Mut/Del). Cells were incubated with Prexasertib for 24 hours and caspase 3/7 activation assessed with the Caspase Glo 3/7 assay system (Promega™). Each point represents the mean of 3 independent experiments. P value was calculated with the paired Student's T test. RLU (relative light units).

B) Graph showing fold changes over time in caspase 3/7 activity in DLBCL cell lines treated with Prexasertib according to the *TP53* status. *TP53* mut/del cell lines showed a tendency towards increased caspase 3/7 activation at late time points (48 hrs).

Error bars represent standard error of the mean (S.E.M) of triplicate experiments. Differences between groups were calculated with the Student T test. * $p < 0.05$, ** $p < 0.01$.

C) Scatter plot representing the effects of 125 nM Prexasertib on apoptosis in DLBCL cell lines according to *BCL-2* status (WT vs Rearranged). Cells were incubated with Prexasertib for 24 hours and caspase 3/7 activation assessed with the Caspase Glo 3/7 assay system (Promega™). Each point represents the mean of 3 independent experiments. P value was calculated with the paired Student's T test. RLU (relative light units).

D) Scatter plot representing the effects of 125 nM Prexasertib on apoptosis in DLBCL cell lines according to *MYC* and *BCL-2* status [*BCL-2* WT vs *BCL-2* Rearranged vs *MYC/BCL-2* rearranged (DH)]. Cells were incubated with Prexasertib for 24 hours and caspase 3/7 activation assessed with the Caspase Glo 3/7 assay system (Promega™). Each point represents the mean of 3 independent experiments. P value was calculated with the paired Student's T test. RLU (relative light units).

E) Scatter plot representing the effects of 125 nM Prexasertib on cell cycle in DLBCL cell lines according to *TP53* status (WT vs Mut/Del). Cells were incubated with Prexasertib for 24 hours and changes in cell cycle fractions assessed by flow cytometry with propidium iodide staining. Each point represents the mean of 3 independent experiments. P value was calculated with the paired Student's T test.

F) Scatter plot representing the effects of 125 nM Prexasertib on cell cycle in DLBCL cell lines according to *BCL-2* status (WT vs Rearranged). Cells were incubated with Prexasertib for 24 hours and changes in cell cycle fractions assessed by flow cytometry with propidium iodide staining. Each point represents the mean of 3 independent experiments. P value was calculated with the paired Student's T test.

Figure S6. HTS data analysis

A) Boxplot graph summarizing the results of the combinatorial drug screening (AZD7762 + Venetoclax) analyzed with the Bliss model in individual cell lines categorized according to the cell of origin and MYC/BCL-2 status. Error bars represent minimum and maximum measured values.

B) Boxplot graph summarizing the results of the combinatorial drug screening (MK8776 + Venetoclax) analyzed with the Bliss model in individual cell lines categorized according to the cell of origin and MYC/BCL-2 status. Error bars represent minimum and maximum measured values.

C) Boxplot graph summarizing the results of the combinatorial drug screening (AZD7762 + Venetoclax) analyzed with the Bliss model according to the cell of origin and BCL-2 status. Error bars represent minimum and maximum measured values. Differences between groups (ABC, GCB *BCL-2 rearranged*, GCB *BCL-2 negative*) were calculated with the Student's t test. *$p < 0.05$, **$p < 0.01$.

D) Boxplot graph summarizing the results of the combinatorial drug screening (MK8776 + Venetoclax) analyzed with the Bliss model according to the cell of origin and BCL-2 status. Error bars represent minimum and maximum measured values. Differences between groups (ABC, GCB *BCL-2 rearranged*, GCB *BCL-2 negative*) were calculated with the Student's t test. *$p < 0.05$, **$p < 0.01$.

E) Western blot assay showing levels of BCL-2 protein expression in the cell line panel used for HTS experiments.

Figure S7. Validation combinatory experiments of Prexasertib and Venetoclax in DLBCL cell lines.

Heat maps showing viability following treatment for 24 hours with increasing doses (D) (12.5-25-50 nM) of Prexasertib (P), Venetoclax (V), and the combination (C), in DLBCL cell lines. Viability values (mean of 3 independent experiments) are depicted in a colorimetric scale from red (low) to yellow (medium) to green (high) with respect to DMSO (control).

Figure S8. Baseline γH2AX and 8-OHdG levels (OX_status), MYC/BCL-2 status, and the activity of combined DDR and BCL-2 inhibition in DLBCL cell lines.

A) Western blot assay showing levels of MYC and BCL-2 protein expression in the cell line panel used for validation experiments.
B) Bar graph showing quantitative densitometry analyses (ImageJ software) of baseline γH2AX levels normalized vs actin (3 independent western blots assays shown in Figure S8C) in our panel of DLBCL cell lines. Error bars represent standard error of the mean (SEM) of triplicate measurements.
C) Three independent western blot assays showing γH2AX protein expression (and actin) in the cell line panel used for validation experiments.
D) Bar graph showing baseline 8-OHdG levels assessed with a dedicated ELISA assay (3 independent measurements) in our panel of DLBCL cell lines. Error bars represent standard error of the mean (SEM) of triplicate measurements.
E) Heat map showing *MYC* and *BCL-2* mRNA levels assessed by qPCR in our cell line panel, γH2AX levels as depicted in panel B, 8-OHdG levels as assessed by ELISA and depicted in panel D [in a colorimetric scale from green (low) to yellow (medium) to red (high)], DE lymphoma (DEL) status based on qPCR or western blot data (in red), and OX_status based on γH2AX/8-OHdG levels. For each individual variable values below the median were considered as “low”, whereas values equal or above

the median were considered as “high”. OX_status was defined as a) OX_H-H (high γH2AX/high 8-OHdG, in red, corresponding to the OX_high status defined in DLBCL tissues by IHC), OX_H-L/L-H (either high γH2AX/low 8-OHdG or low γH2AX/high 8-OHdG, in orange) and OX_L-L (low γH2AX/low 8-OHdG, in blue). The latter two categories correspond to the OX_low status defined by IHC.

F) Cell titer glo assays of DLBCL cell lines treated with single agent Venetoclax and Prexasertib and classified by combining DE status evaluated by western blot (MYC and BCL-2 protein levels) with γH2AX/8-OHdG status (based on the heat map showed in panel D): cell lines were classified as a) DE-OX_H-H (high MYC/BCL-2, high γH2AX/high 8-OHdG, in red), DE-OX_H-L/L-H (high MYC/BCL-2, and either high γH2AX/low 8-OHdG or low γH2AX/high 8-OHdG, in orange) and DE-OX_L-L (high MYC/BCL-2, low γH2AX/low 8-OHdG, in blue). Cells were incubated with increasing doses of Prexasertib or Venetoclax for 24 hours (12.5-25-50 nM). Viability values represent the mean of 3 independent experiments.

G) Scatterplot representing combination index data of Prexasertib combined with Venetoclax in 9 DE DLBCL cell lines (as defined by MYC and BCL-2 protein levels) according to the baseline γH2AX/8-OHdG (OX) status (H-H in red vs L-L in blue vs L-H/H-L in orange). 3 doses of Prexasertib (12.5, 25, 50 nM) were combined with 3 doses of Venetoclax (12.5, 25, 50 nM) for 24 hours, allowing 3 combinatory values for each cell line. Values below 1 indicate synergistic interactions.See also figure 4C.

Figure S9. DDR inhibition and DDRi-based combinations in BCL-2 negative cell lines

A) Western blot assays showing the effects of 24 hours incubation with Prexasertib 125 nM, Doxorubicin 125 nM and H2O2 150 µM, on BCL-2 and γH2AX levels in 2 BCL-2 negative (SUDHL-5 and OCILY-7) and one BCL-2 positive cell line (HBL-1).
B) Cell Titer Glo experiments in 2 representative BCL-2 negative (SUDHL-5, SUDHL-2), and one BCL-2 positive (TMD8) cell line. Cells were incubated with increasing concentrations of Prexasertib and the MCL-1 inhibitor S63845 (12.5, 25, 50 nM), and cell viability was assessed after 24 hours. Error bars represent standard error of the mean (S.E.M) of triplicate experiments.

Figure S10. Role of BCL-2 in modulating sensitivity to DDR inhibition.

A) Experimental design of the experiments represented in Figure 5 A-D. SUDHL-5 cells (transfected with EMPTY VECTOR or a BCL-2 TET-ON inducible system (BCL-2 EXP) were preincubated with doxycycline 1 µg/ml for 24 hours and then treated with Prexasertib at the indicated doses. Lower panel: Growth curves of SUDHL-5 cells cultured in the presence or absence of BCL-2 (EMPTY vs BCL-2) for up to 96 hrs.
B) Western blot assays from triplicate experiments depicting the effects of Prexasertib treatment (6nM) on γH2AX induction in the presence or absence of BCL-2 in SUDHL-5 cells. Densitometry analyses were performed with the ImageJ software and are represented in Figure 5B.

Figure S11. Role of MYC in modulating sensitivity to DDR inhibition.

A) Experimental design of the experiments represented in Figure 5 E-H. P-4936 cells (carrying a tetracycline inducible promoter, TET-OFF MYC) were preincubated with doxycycline 1 µg/ml for 6 hours and then treated with Prexasertib at the indicated

doses. Lower panel: Growth curves of P-4936 cells cultured in the presence or absence of MYC (MYC-ON vs MYC-OFF) for up to 96 hrs.

B) Western blot assays from triplicate experiments depicting the effects of Prexasertib treatment (25 nM) on γH2AX induction in the presence or absence of MYC in P-4936 cells. Densitometry analyses were performed with the ImageJ software and are represented in Figure 5F.

C) Left panel: Western blot assays from triplicate experiments depicting the effects of MYC depletion on baseline γH2AX levels in P-4936 cells. Densitometry analyses of γH2AX (based on the blots represented in the left panel) were performed with the ImageJ software and are represented in the right panel.

Figure S12. Variations in body weight over time in mice treated with Prexasertib, Venetoclax and the combinations in a PDX DH-DLBCL mouse model.

A) Graph showing variations of body weight over time in mice treated with vehicle (n=5) Prexasertib 1.25 and 2.5 mg/kg BID 3 times/week (n=5), Venetoclax 50 mg/Kg 5 days a week (n=5), and the combinations (n=5).

B) Columns graphs showing body weights at day 41 in mice treated with vehicle, prexasertib (1.25 and 2.5 mg/Kg 3 times/week), venetoclax 50 mg/Kg 5 days a week, and the combinations.

References

1- Chiappella A, Martelli M, Angelucci E, Brusamolino E, Evangelista A, Carella AM et al. Rituximab-dose-dense chemotherapy with or without high-dose chemotherapy plus autologous stem-cell transplantation in high-risk diffuse large B-cell lymphoma (DLCL04): final results of a multicentre, open-label, randomised, controlled, phase 3 study. Lancet Oncol. 2017; 18(8): 1076-1088.

2- Swerdlow SH, Campo E, Harris NL, Jaffe ES, Pileri SA, Stein H et al. WHO Classification of Tumours of the Haematopoietic and Lymphoid Tissues. Revised 4th ed Lyon, France: IARC; 2017.

3- Went P, Agostinelli C, Gallamini A, Piccaluga PP, Ascani S, Sabattini E, et al. Marker expression in peripheral T-cell lymphoma: a proposed clinical-pathologic prognostic score. J Clin Oncol. 2006; 24:2472–2479.

4- Cerami E, Gao J, Dogrusoz U, Gross BE, Sumer SO, Aksoy BA, et al. The cBio cancer genomics portal: an open platform for exploring multidimensional cancer genomics data. Cancer Discov. 2012;2:401-4.

5- Derenzini E, Agostinelli C, Imbrogno E, Iacobucci I, Casadei B, Brighenti E et al. Constitutive activation of the DNA damage response pathway as a novel therapeutic target in diffuse large B-cell lymphoma. Oncotarget. 2015;6:6553-69.

6- Juskevicius D, Müller A, Hashwah H, Lundberg P, Tzankov A, Menter T. Characterization of the mutational profile of 11 diffuse large B-cell lymphoma cell lines. Leuk Lymphoma. 2018;59:1710-1716.

7- Hicks SW, Tarantelli C, Wilhem A, Gaudio E, Li M, Arribas AJ, Spriano F, et al. The novel CD19-targeting antibody-drug conjugate huB4-DGN462 shows improved anti-tumor activity compared to SAR3419 in CD19-positive lymphoma and leukemia models. Haematologica. 2019;104:1633-1639.

8- Li W, Gupta SK, Han W, Kundson RA, Nelson S, Knutson D, et al. Targeting MYC activity in double-hit lymphoma with MYC and BCL2 and/or BCL6 rearrangements with epigenetic bromodomain inhibitors. J Hematol Oncol. 2019;12:73.

9- Scott DW, Wright GW, Williams PM, Lih CJ, Walsh W, Jaffe ES et al. Determining cell-of-origin subtypes of diffuse large B-cell lymphoma using gene expression in formalin-fixed paraffin-embedded tissue. Blood. 2014; 123(8): 1214-7.

10- Derenzini E, Mondello P, Erazo T, Portelinha A, Liu Y, Scallion M et al. BET Inhibition-Induced GSK3β Feedback Enhances Lymphoma Vulnerability to PI3K Inhibitors. Cell Rep. 2018; 24(8): 2155-2166.

11- Feller W. An Introduction to Probability Theory and Its Applications. John Wiley&Sons, New York, NY (1971).

12- Tallarida RJ. Drug synergism: its detection and applications. J Pharmacol Exp Ther. 2001 Sep;298(3):865-72.

13- R Core Team. R: A language and environment for statistical computing. R Foundation for Statistical Computing, Vienna, Austria. 2014. URL http://www.R-project.org/.

14- Chou TC. Drug combination studies and their synergy quantification using the Chou-Talalay method. Cancer Res. 2010;70:440-6.

HIGH THROUGHPUT DRUG SCREENING (HTS)

DATA SUPPLEMENT

AZD7762 + ABT-199 (VENETOCLAX)

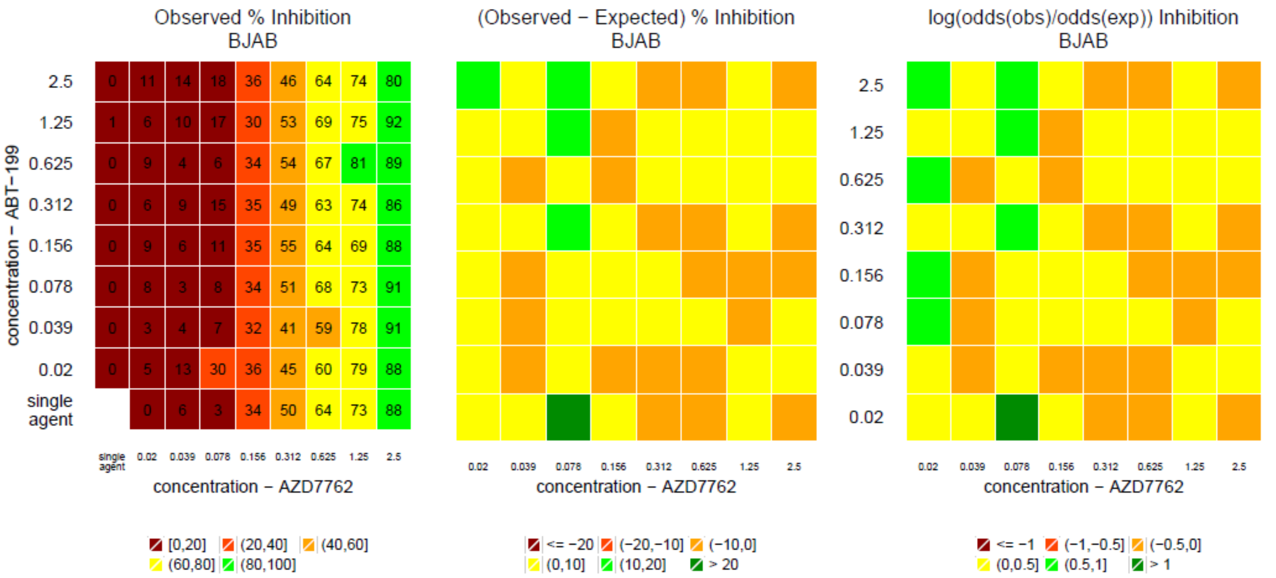

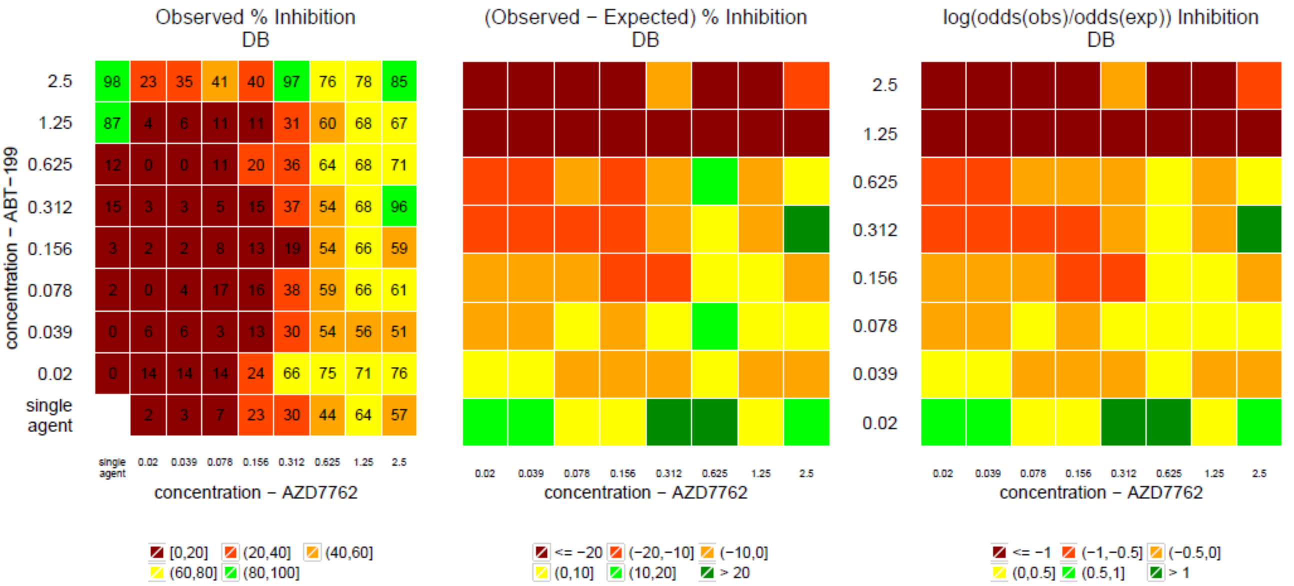

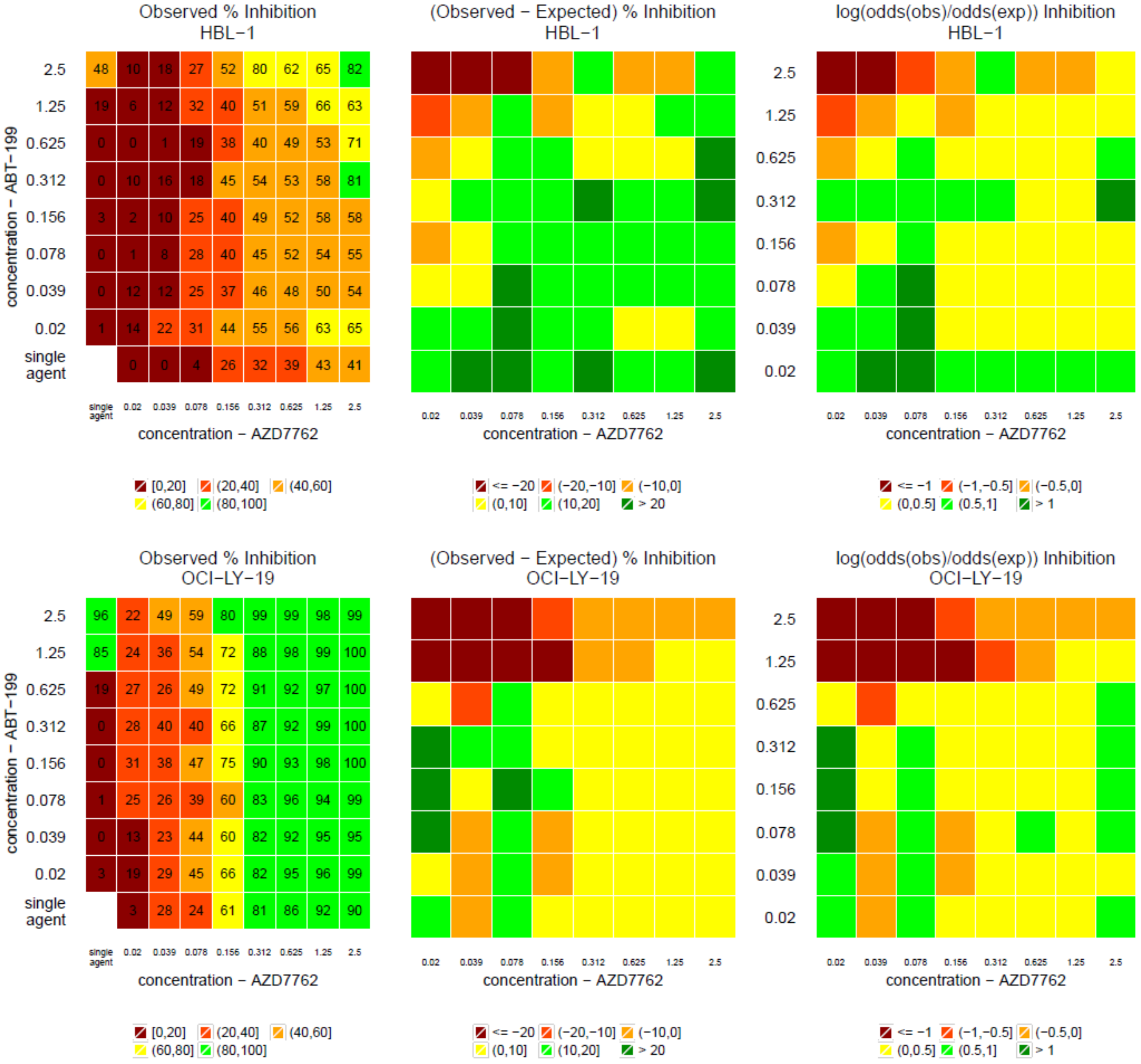
Observed % Inhibition
HBL−1
concentration − ABT−199
2.5 | 48 10 18 27 52 80 62 65 82
1.25 | 19 6 12 32 40 51 59 66 63
0.625 | 0 0 1 19 38 40 49 53 71
0.312 | 0 10 16 18 45 54 53 58 81
0.156 | 3 2 10 25 40 49 52 58 58
0.078 | 0 1 8 28 40 45 52 54 55
0.039 | 0 12 12 25 37 46 48 50 54
0.02 | 1 14 22 31 44 55 56 63 65
single agent | 0 0 4 26 32 39 43 41
single agent 0.02 0.039 0.078 0.156 0.312 0.625 1.25 2.5
concentration − AZD7762
[0,20] (20,40] (40,60] (60,80] (80,100]
(Observed − Expected) % Inhibition
HBL−1
2.5 1.25 0.625 0.312 0.156 0.078 0.039 0.02
0.02 0.039 0.078 0.156 0.312 0.625 1.25 2.5
concentration − AZD7762
<= −20 (−20,−10] (−10,0] (0,10] (10,20] > 20
log(odds(obs)/odds(exp)) Inhibition
HBL−1
2.5 1.25 0.625 0.312 0.156 0.078 0.039 0.02
0.02 0.039 0.078 0.156 0.312 0.625 1.25 2.5
concentration − AZD7762
<= −1 (−1,−0.5] (−0.5,0] (0,0.5] (0.5,1] > 1
Observed % Inhibition
OCI−LY−19
concentration − ABT−199
2.5 | 96 22 49 59 80 99 99 98 99
1.25 | 85 24 36 54 72 88 98 99 100
0.625 | 19 27 26 49 72 91 92 97 100
0.312 | 0 28 40 40 66 87 92 99 100
0.156 | 0 31 38 47 75 90 93 98 100
0.078 | 1 25 26 39 60 83 96 94 99
0.039 | 0 13 23 44 60 82 92 95 95
0.02 | 3 19 29 45 66 82 95 96 99
single agent | 3 28 24 61 81 86 92 90
single agent 0.02 0.039 0.078 0.156 0.312 0.625 1.25 2.5
concentration − AZD7762
[0,20] (20,40] (40,60] (60,80] (80,100]
(Observed − Expected) % Inhibition
OCI−LY−19
2.5 1.25 0.625 0.312 0.156 0.078 0.039 0.02
0.02 0.039 0.078 0.156 0.312 0.625 1.25 2.5
concentration − AZD7762
<= −20 (−20,−10] (−10,0] (0,10] (10,20] > 20
log(odds(obs)/odds(exp)) Inhibition
OCI−LY−19
2.5 1.25 0.625 0.312 0.156 0.078 0.039 0.02
0.02 0.039 0.078 0.156 0.312 0.625 1.25 2.5
concentration − AZD7762
<= −1 (−1,−0.5] (−0.5,0] (0,0.5] (0.5,1] > 1

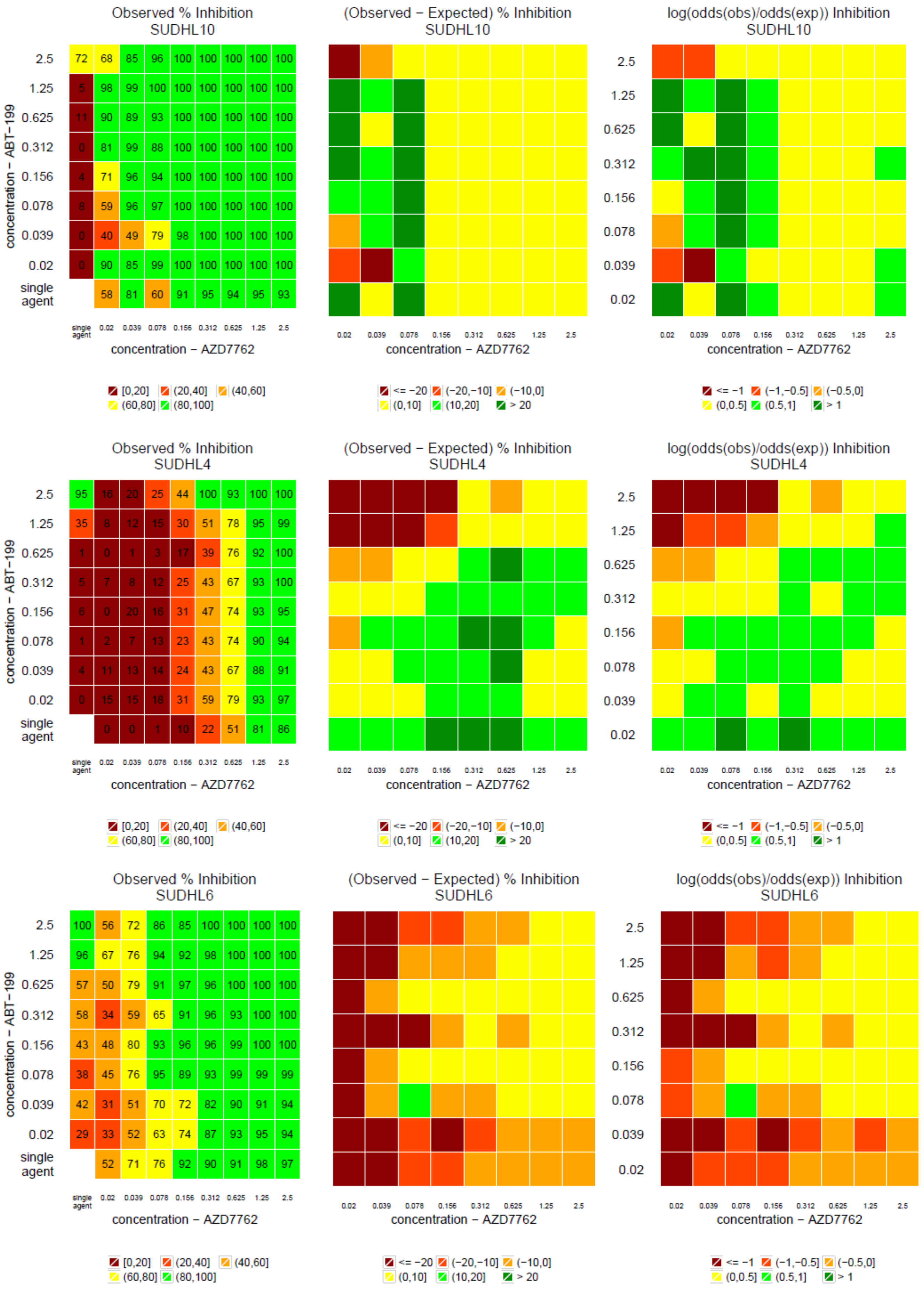

Observed % Inhibition SUDHL10
2.5 72 68 85 96 100 100 100 100 100
1.25 5 98 99 100 100 100 100 100 100
0.625 11 90 89 93 100 100 100 100 100
0.312 0 81 99 88 100 100 100 100 100
0.156 4 71 96 94 100 100 100 100 100
0.078 8 59 96 97 100 100 100 100 100
0.039 0 40 49 79 98 100 100 100 100
0.02 0 90 85 99 100 100 100 100 100
single agent 58 81 60 91 95 94 95 93
single agent 0.02 0.039 0.078 0.156 0.312 0.625 1.25 2.5
concentration − ABT−199
concentration − AZD7762
[0,20] (20,40] (40,60] (60,80] (80,100]
(Observed − Expected) % Inhibition SUDHL10
<= −20 (−20,−10] (−10,0] (0,10] (10,20] > 20
log(odds(obs)/odds(exp)) Inhibition SUDHL10
<= −1 (−1,−0.5] (−0.5,0] (0,0.5] (0.5,1] > 1
Observed % Inhibition SUDHL4
2.5 95 16 20 25 44 100 93 100 100
1.25 35 8 12 15 30 51 78 95 99
0.625 1 0 1 3 17 39 76 92 100
0.312 5 7 8 12 25 43 67 93 100
0.156 6 0 20 16 31 47 74 93 95
0.078 1 2 7 13 23 43 74 90 94
0.039 4 11 13 14 24 43 67 88 91
0.02 0 15 15 18 31 59 79 93 97
single agent 0 0 1 10 22 51 81 86
(Observed − Expected) % Inhibition SUDHL4
log(odds(obs)/odds(exp)) Inhibition SUDHL4
Observed % Inhibition SUDHL6
2.5 100 56 72 86 85 100 100 100 100
1.25 96 67 76 94 92 98 100 100 100
0.625 57 50 79 91 97 96 100 100 100
0.312 58 34 59 65 91 96 93 100 100
0.156 43 48 80 93 96 96 99 100 100
0.078 38 45 76 95 89 93 99 99 99
0.039 42 31 51 70 72 82 90 91 94
0.02 29 33 52 63 74 87 93 95 94
single agent 52 71 76 92 90 91 98 97
(Observed − Expected) % Inhibition SUDHL6
log(odds(obs)/odds(exp)) Inhibition SUDHL6

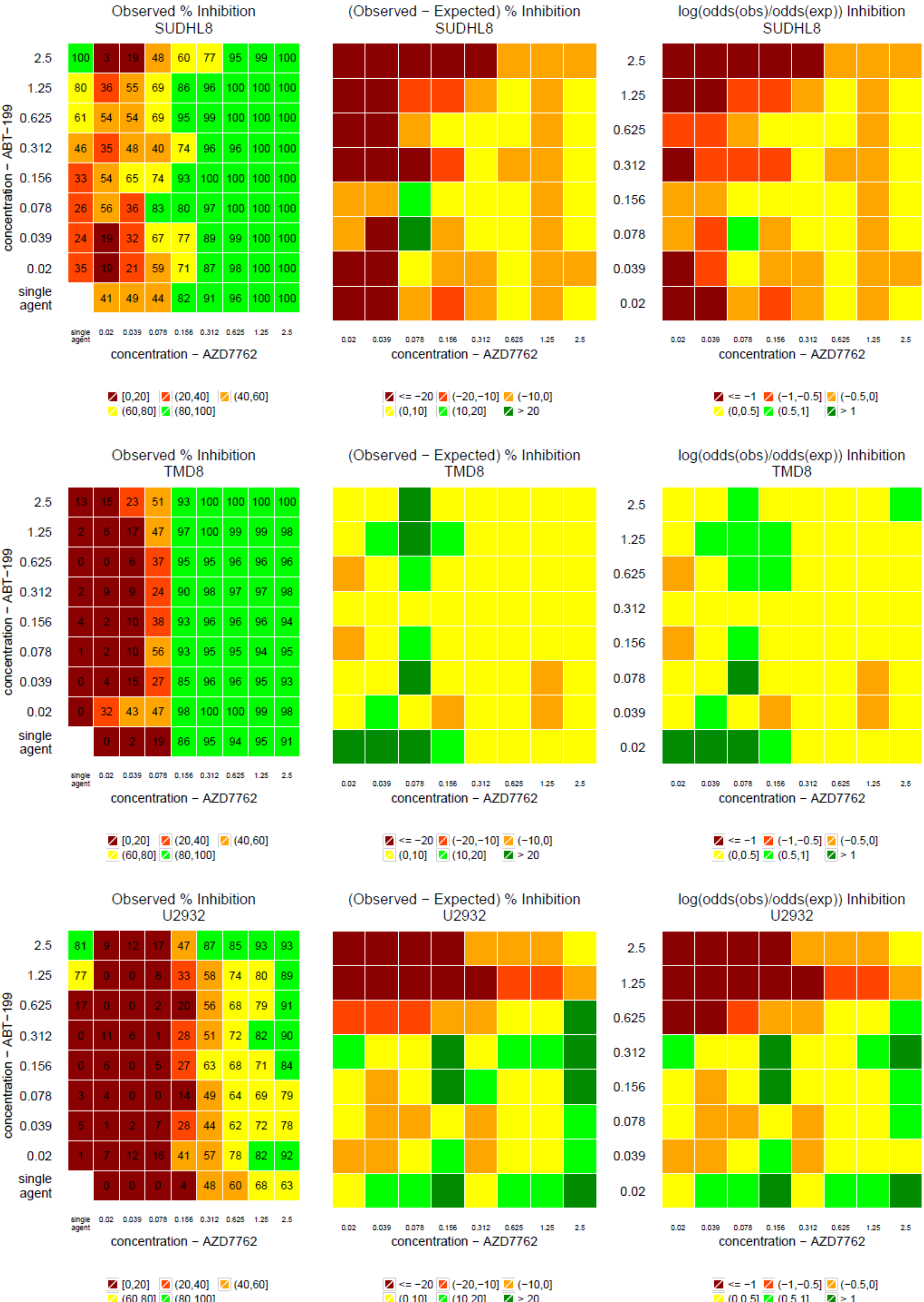
Observed % Inhibition
SUDHL8
(Observed − Expected) % Inhibition
SUDHL8
log(odds(obs)/odds(exp)) Inhibition
SUDHL8
concentration − ABT−199
concentration − AZD7762
[0,20] (20,40] (40,60] (60,80] (80,100]
<= −20 (−20,−10] (−10,0] (0,10] (10,20] > 20
<= −1 (−1,−0.5] (−0.5,0] (0,0.5] (0.5,1] > 1
Observed % Inhibition
TMD8
(Observed − Expected) % Inhibition
TMD8
log(odds(obs)/odds(exp)) Inhibition
TMD8
concentration − ABT−199
concentration − AZD7762
[0,20] (20,40] (40,60] (60,80] (80,100]
<= −20 (−20,−10] (−10,0] (0,10] (10,20] > 20
<= −1 (−1,−0.5] (−0.5,0] (0,0.5] (0.5,1] > 1
Observed % Inhibition
U2932
(Observed − Expected) % Inhibition
U2932
log(odds(obs)/odds(exp)) Inhibition
U2932
concentration − ABT−199
concentration − AZD7762
[0,20] (20,40] (40,60] (60,80] (80,100]
<= −20 (−20,−10] (−10,0] (0,10] (10,20] > 20
<= −1 (−1,−0.5] (−0.5,0] (0,0.5] (0.5,1] > 1

MK8776 + ABT-199 (VENETOCLAX)

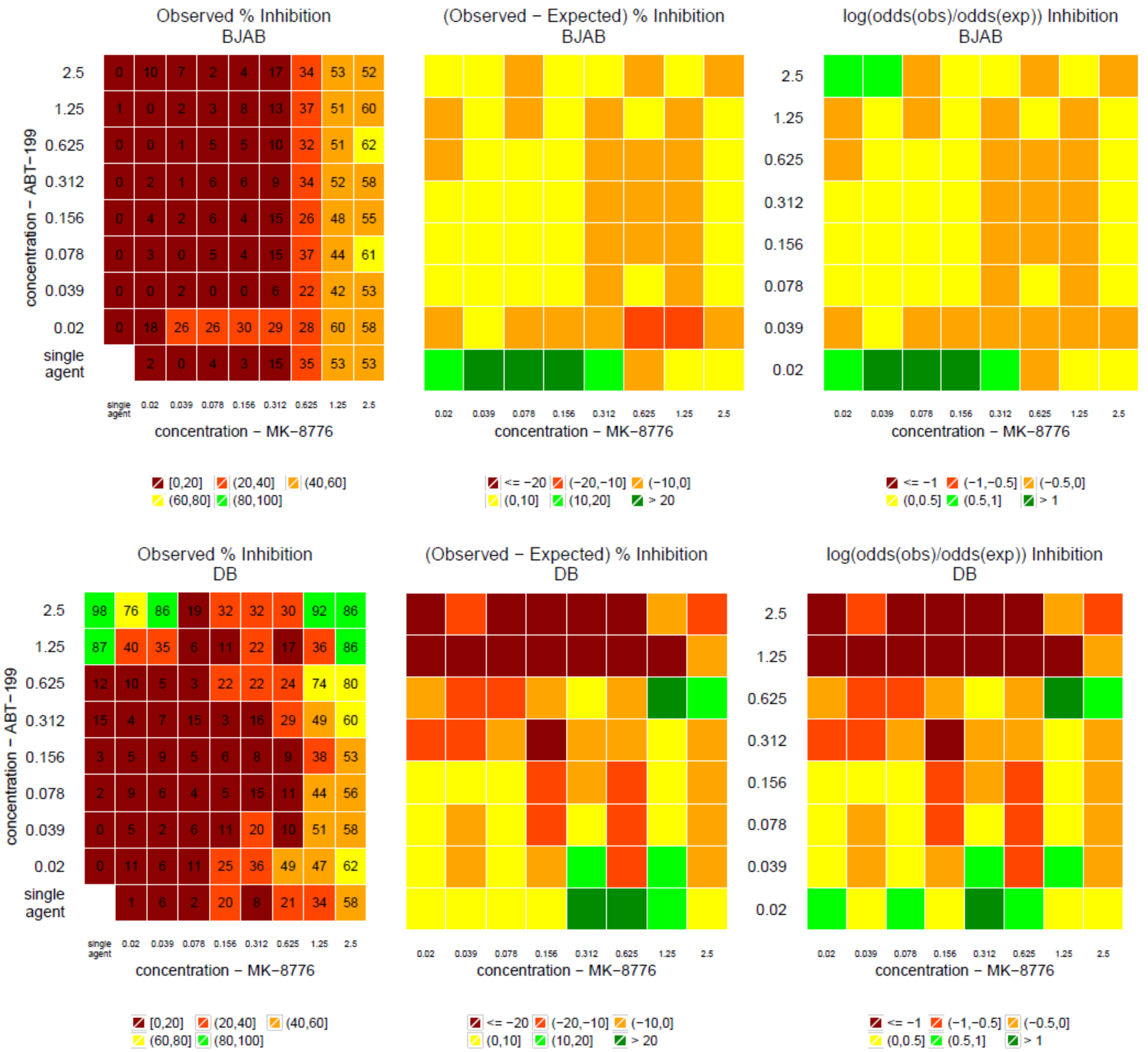

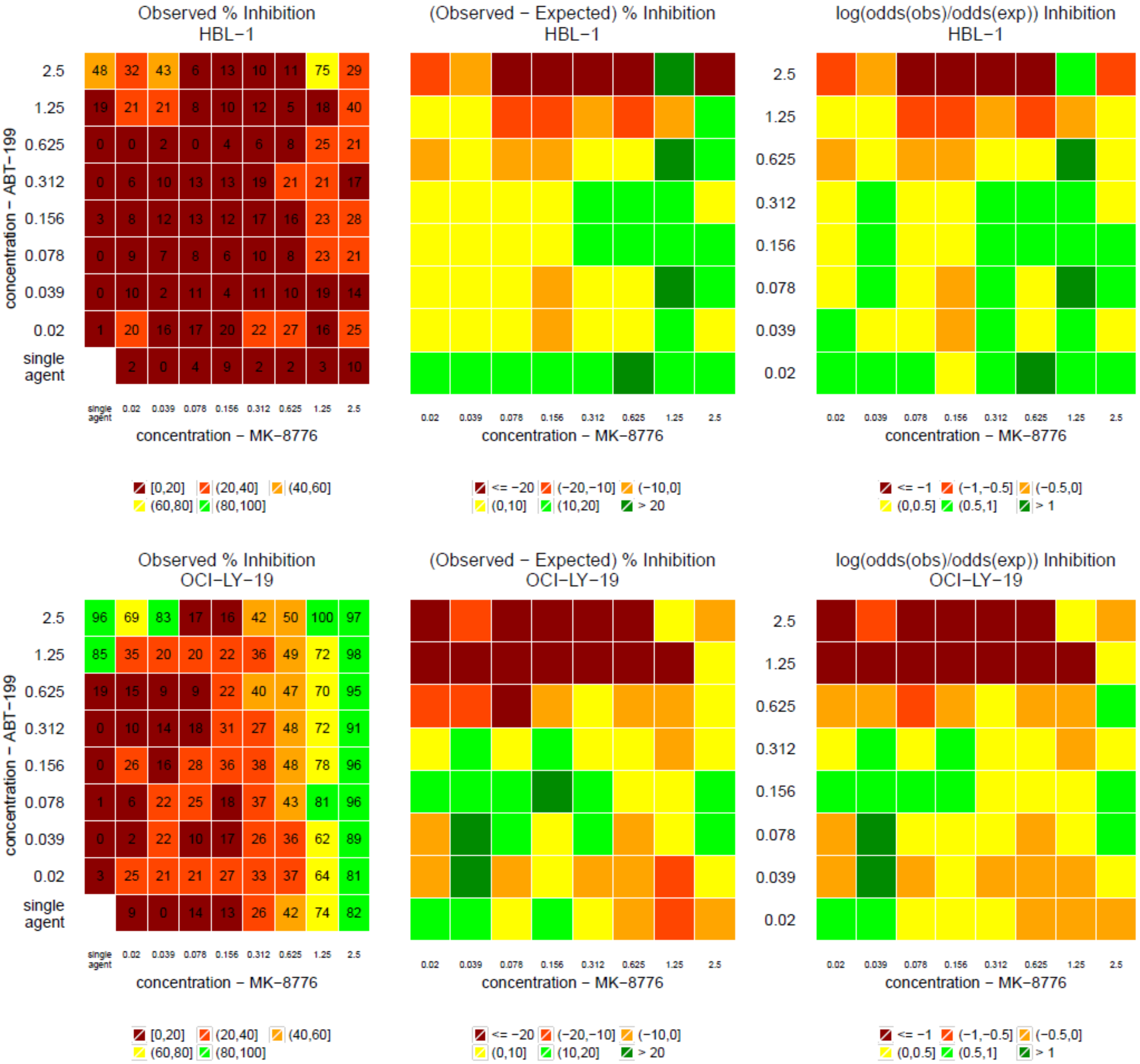
Observed % Inhibition
HBL−1
2.5: 48 32 43 6 13 10 11 75 29
1.25: 19 21 21 8 10 12 5 18 40
0.625: 0 0 2 0 4 6 8 25 21
0.312: 0 6 10 13 13 19 21 21 17
0.156: 3 8 12 13 12 17 16 23 28
0.078: 0 9 7 8 6 10 8 23 21
0.039: 0 10 2 11 4 11 10 19 14
0.02: 1 20 16 17 20 22 27 16 25
single agent: 2 0 4 9 2 2 3 10
concentration − ABT−199
single agent 0.02 0.039 0.078 0.156 0.312 0.625 1.25 2.5
concentration − MK−8776
[0,20] (20,40] (40,60] (60,80] (80,100]
(Observed − Expected) % Inhibition
HBL−1
2.5 1.25 0.625 0.312 0.156 0.078 0.039 0.02
0.02 0.039 0.078 0.156 0.312 0.625 1.25 2.5
concentration − MK−8776
<= −20 (−20,−10] (−10,0] (0,10] (10,20] > 20
log(odds(obs)/odds(exp)) Inhibition
HBL−1
2.5 1.25 0.625 0.312 0.156 0.078 0.039 0.02
0.02 0.039 0.078 0.156 0.312 0.625 1.25 2.5
concentration − MK−8776
<= −1 (−1,−0.5] (−0.5,0] (0,0.5] (0.5,1] > 1
Observed % Inhibition
OCI−LY−19
2.5: 96 69 83 17 16 42 50 100 97
1.25: 85 35 20 20 22 36 49 72 98
0.625: 19 15 9 9 22 40 47 70 95
0.312: 0 10 14 18 31 27 48 72 91
0.156: 0 26 16 28 36 38 48 78 96
0.078: 1 6 22 25 18 37 43 81 96
0.039: 0 2 22 10 17 26 36 62 89
0.02: 3 25 21 21 27 33 37 64 81
single agent: 9 0 14 13 26 42 74 82
concentration − ABT−199
single agent 0.02 0.039 0.078 0.156 0.312 0.625 1.25 2.5
concentration − MK−8776
[0,20] (20,40] (40,60] (60,80] (80,100]
(Observed − Expected) % Inhibition
OCI−LY−19
0.02 0.039 0.078 0.156 0.312 0.625 1.25 2.5
concentration − MK−8776
<= −20 (−20,−10] (−10,0] (0,10] (10,20] > 20
log(odds(obs)/odds(exp)) Inhibition
OCI−LY−19
2.5 1.25 0.625 0.312 0.156 0.078 0.039 0.02
0.02 0.039 0.078 0.156 0.312 0.625 1.25 2.5
concentration − MK−8776
<= −1 (−1,−0.5] (−0.5,0] (0,0.5] (0.5,1] > 1

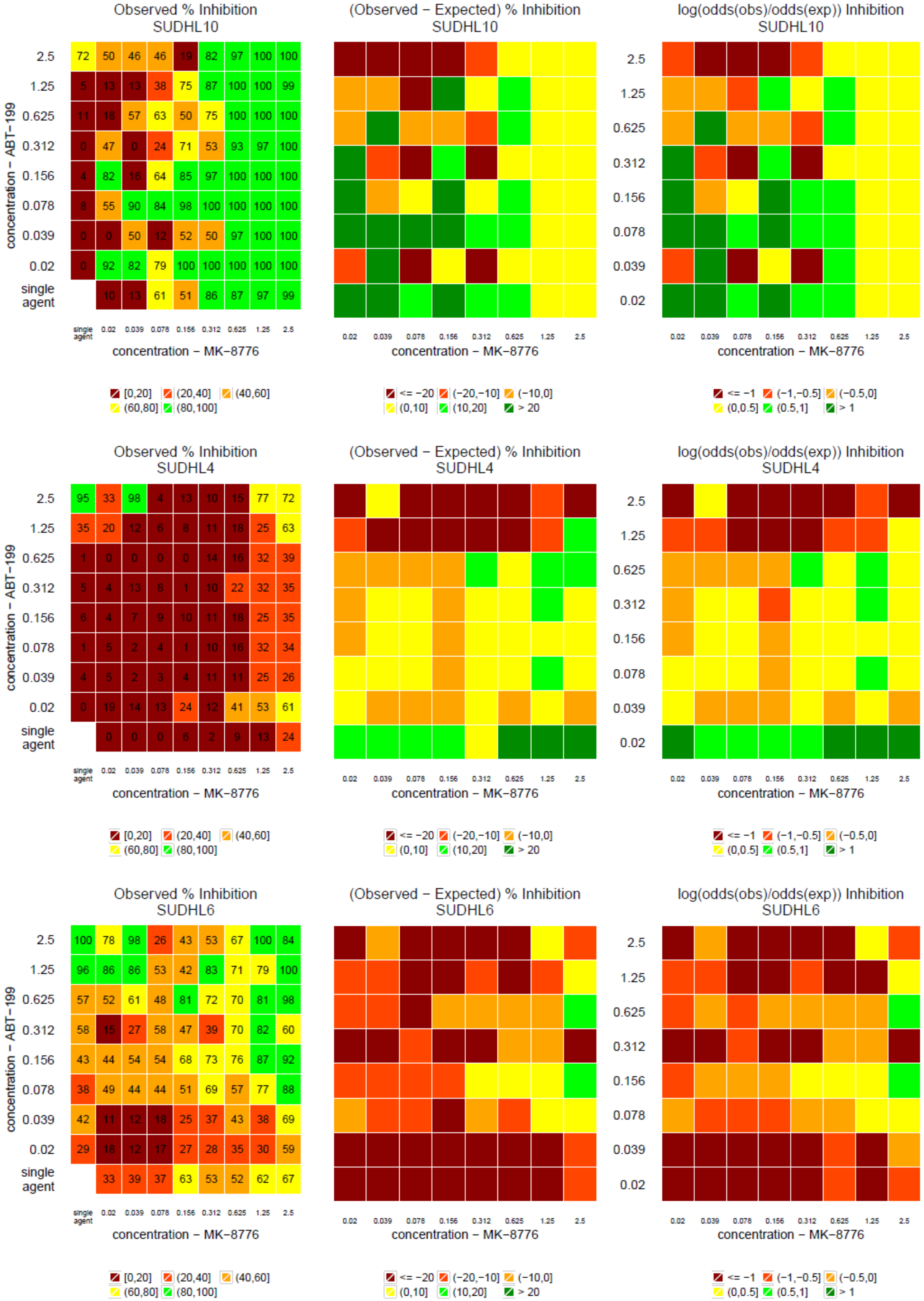
Observed % Inhibition
SUDHL10
(Observed − Expected) % Inhibition
SUDHL10
log(odds(obs)/odds(exp)) Inhibition
SUDHL10
concentration − ABT−199
2.5 72 50 46 46 19 82 97 100 100
1.25 5 13 13 38 75 87 100 100 99
0.625 11 18 57 63 50 75 100 100 100
0.312 0 47 0 24 71 53 93 97 100
0.156 4 82 16 64 85 97 100 100 100
0.078 8 55 90 84 98 100 100 100 100
0.039 0 0 50 12 52 50 97 100 100
0.02 0 92 82 79 100 100 100 100 100
single agent 10 13 61 51 86 87 97 99
single agent 0.02 0.039 0.078 0.156 0.312 0.625 1.25 2.5
concentration − MK−8776
[0,20] (20,40] (40,60] (60,80] (80,100]
<= −20 (−20,−10] (−10,0] (0,10] (10,20] > 20
<= −1 (−1,−0.5] (−0.5,0] (0,0.5] (0.5,1] > 1
Observed % Inhibition
SUDHL4
(Observed − Expected) % Inhibition
SUDHL4
log(odds(obs)/odds(exp)) Inhibition
SUDHL4
2.5 95 33 98 4 13 10 15 77 72
1.25 35 20 12 6 8 11 18 25 63
0.625 1 0 0 0 0 14 16 32 39
0.312 5 4 13 8 1 10 22 32 35
0.156 6 4 7 9 10 11 18 25 35
0.078 1 5 2 4 1 10 16 32 34
0.039 4 5 2 3 4 11 11 25 26
0.02 0 19 14 13 24 12 41 53 61
single agent 0 0 0 6 2 9 13 24
Observed % Inhibition
SUDHL6
(Observed − Expected) % Inhibition
SUDHL6
log(odds(obs)/odds(exp)) Inhibition
SUDHL6
2.5 100 78 98 26 43 53 67 100 84
1.25 96 86 86 53 42 83 71 79 100
0.625 57 52 61 48 81 72 70 81 98
0.312 58 15 27 58 47 39 70 82 60
0.156 43 44 54 54 68 73 76 87 92
0.078 38 49 44 44 51 69 57 77 88
0.039 42 11 12 18 25 37 43 38 69
0.02 29 18 12 17 27 28 35 30 59
single agent 33 39 37 63 53 52 62 67

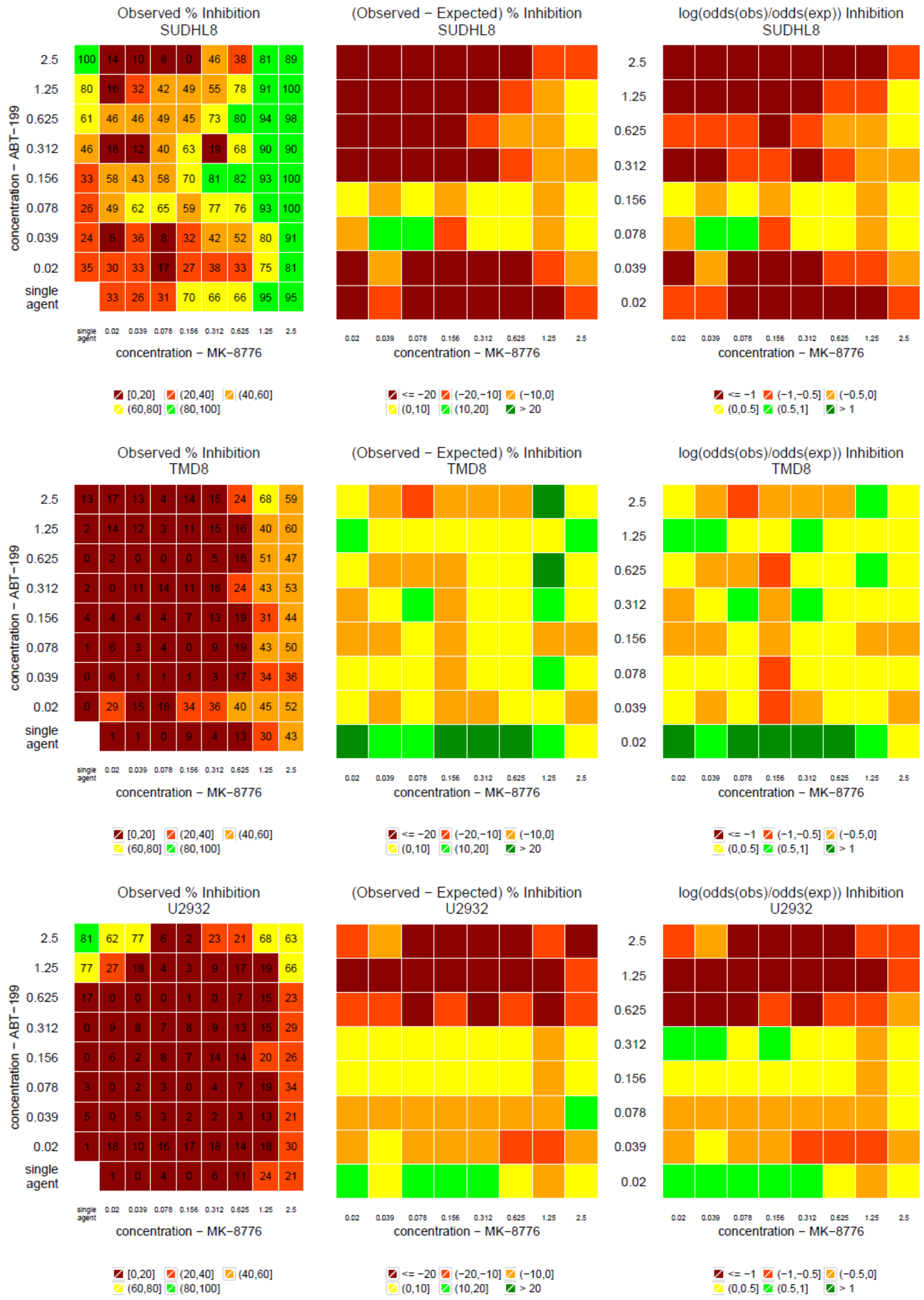
Observed % Inhibition
SUDHL8
concentration − ABT−199
2.5 100 14 10 8 0 46 38 81 89
1.25 80 16 32 42 49 55 78 91 100
0.625 61 46 46 49 45 73 80 94 98
0.312 46 18 12 40 63 19 68 90 90
0.156 33 58 43 58 70 81 82 93 100
0.078 26 49 62 65 59 77 76 93 100
0.039 24 6 36 8 32 42 52 80 91
0.02 35 30 33 17 27 38 33 75 81
single agent 33 26 31 70 66 66 95 95
single agent 0.02 0.039 0.078 0.156 0.312 0.625 1.25 2.5
concentration − MK−8776
[0,20] (20,40] (40,60] (60,80] (80,100]
(Observed − Expected) % Inhibition
SUDHL8
0.02 0.039 0.078 0.156 0.312 0.625 1.25 2.5
concentration − MK−8776
<= −20 (−20,−10] (−10,0] (0,10] (10,20] > 20
log(odds(obs)/odds(exp)) Inhibition
SUDHL8
2.5 1.25 0.625 0.312 0.156 0.078 0.039 0.02
0.02 0.039 0.078 0.156 0.312 0.625 1.25 2.5
concentration − MK−8776
<= −1 (−1,−0.5] (−0.5,0] (0,0.5] (0.5,1] > 1
Observed % Inhibition
TMD8
concentration − ABT−199
2.5 13 17 13 4 14 15 24 68 59
1.25 2 14 12 3 11 15 16 40 60
0.625 0 2 0 0 0 5 16 51 47
0.312 2 0 11 14 11 16 24 43 53
0.156 4 4 4 4 7 13 19 31 44
0.078 1 6 3 4 0 9 19 43 50
0.039 0 6 1 1 1 3 17 34 36
0.02 0 29 15 18 34 36 40 45 52
single agent 1 1 0 9 4 13 30 43
single agent 0.02 0.039 0.078 0.156 0.312 0.625 1.25 2.5
concentration − MK−8776
[0,20] (20,40] (40,60] (60,80] (80,100]
(Observed − Expected) % Inhibition
TMD8
0.02 0.039 0.078 0.156 0.312 0.625 1.25 2.5
concentration − MK−8776
<= −20 (−20,−10] (−10,0] (0,10] (10,20] > 20
log(odds(obs)/odds(exp)) Inhibition
TMD8
2.5 1.25 0.625 0.312 0.156 0.078 0.039 0.02
0.02 0.039 0.078 0.156 0.312 0.625 1.25 2.5
concentration − MK−8776
<= −1 (−1,−0.5] (−0.5,0] (0,0.5] (0.5,1] > 1
Observed % Inhibition
U2932
concentration − ABT−199
2.5 81 62 77 6 2 23 21 68 63
1.25 77 27 18 4 3 9 17 19 66
0.625 17 0 0 0 1 0 7 15 23
0.312 0 9 8 7 8 9 13 15 29
0.156 0 6 2 8 7 14 14 20 26
0.078 3 0 2 3 0 4 7 19 34
0.039 5 0 5 3 2 2 3 13 21
0.02 1 18 10 16 17 18 14 18 30
single agent 1 0 4 0 6 11 24 21
single agent 0.02 0.039 0.078 0.156 0.312 0.625 1.25 2.5
concentration − MK−8776
[0,20] (20,40] (40,60] (60,80] (80,100]
(Observed − Expected) % Inhibition
U2932
0.02 0.039 0.078 0.156 0.312 0.625 1.25 2.5
concentration − MK−8776
<= −20 (−20,−10] (−10,0] (0,10] (10,20] > 20
log(odds(obs)/odds(exp)) Inhibition
U2932
2.5 1.25 0.625 0.312 0.156 0.078 0.039 0.02
0.02 0.039 0.078 0.156 0.312 0.625 1.25 2.5
concentration − MK−8776
<= −1 (−1,−0.5] (−0.5,0] (0,0.5] (0.5,1] > 1
